# The Misinformation Susceptibility Test (MIST): A psychometrically validated measure of news veracity discernment

**DOI:** 10.3758/s13428-023-02124-2

**Published:** 2023-06-29

**Authors:** Rakoen Maertens, Friedrich M. Götz, Hudson F. Golino, Jon Roozenbeek, Claudia R. Schneider, Yara Kyrychenko, John R. Kerr, Stefan Stieger, William P. McClanahan, Karly Drabot, James He, Sander van der Linden

**Affiliations:** 1 Department of Psychology, University of Cambridge, Downing Street, CB2 3EB Cambridge, Cambridgeshire UK; 2 Department of Psychology, University of British Columbia, 2136 West Mall, Vancouver, BC V6T 1Z4 Canada; 3 University of Virginia, Charlottesville, VA USA; 4 Karl Landsteiner University of Health Sciences, Krems an der Donau, Austria; 5 Max Planck Institute for the Study of Crime, Security and Law, Freiburg im Breisgau, Germany

**Keywords:** Misinformation susceptibility, Automated item generation, Fake news, Neural networks, Psychometrics

## Abstract

Interest in the psychology of misinformation has exploded in recent years. Despite ample research, to date there is no validated framework to measure misinformation susceptibility. Therefore, we introduce ***V****e****r****i****f****ication* ***d****o****n****e*, a nuanced interpretation schema and assessment tool that simultaneously considers ***V****eracity discernment*, and its distinct, measurable abilities (***r****eal/****f****ake news detection*), and biases (***d****istrust*/***n****aïvité*—negative/positive judgment bias). We then conduct three studies with seven independent samples (*N*_total_ = 8504) to show how to develop, validate, and apply the Misinformation Susceptibility Test (MIST). In Study 1 (*N =* 409) we use a neural network language model to generate items, and use three psychometric methods—factor analysis, item response theory, and exploratory graph analysis—to create the MIST-20 (20 items; completion time < 2 minutes), the MIST-16 (16 items; < 2 minutes), and the MIST-8 (8 items; < 1 minute). In Study 2 (*N =* 7674) we confirm the internal and predictive validity of the MIST in five national quota samples (US, UK), across 2 years, from three different sampling platforms—Respondi, CloudResearch, and Prolific. We also explore the MIST’s nomological net and generate age-, region-, and country-specific norm tables. In Study 3 (*N =* 421) we demonstrate how the MIST—in conjunction with ***V****e****r****i****f****ication* ***d****o****n****e*—can provide novel insights on existing psychological interventions, thereby advancing theory development. Finally, we outline the versatile implementations of the MIST as a screening tool, covariate, and intervention evaluation framework. As all methods are transparently reported and detailed, this work will allow other researchers to create similar scales or adapt them for any population of interest.

**Supplementary Information:**

The online version contains supplementary material available at 10.3758/s13428-023-02124-2.

The global spread of misinformation has had a palpable negative impact on society. For instance, conspiracy theories about the coronavirus disease 2019 (COVID-19) vaccines have been linked to increased vaccine hesitancy and a decline in vaccination intentions (Hotez et al., 2021; Loomba et al., 2021; Roozenbeek et al., 2020). Misinformation about the impact of 5G has led to the vandalization of cell phone masts (Jolley & Paterson, 2020), and misinformation about climate change has been associated with a reduction in perceptions of scientific consensus (Maertens et al., 2020; van der Linden et al., 2017). With false and moral-emotional media spreading faster and deeper than more accurate and nuanced content (Brady et al., 2017; Vosoughi et al., 2018), the importance of information veracity has become a central debate for scholars and policymakers (Lewandowsky et al., 2017, 2020).^1^

Accordingly, across disciplines, research on the processes behind, impact of, and interventions against misinformation has surged over the past years (for recent reviews, see Pennycook & Rand, 2021; Roozenbeek et al., 2023; Van Bavel, Harris, et al., 2020; van der Linden et al., 2021). Researchers have made progress in designing media and information literacy interventions in the form of educational games (Basol et al., 2021; Roozenbeek & van der Linden, 2019, 2020), “accuracy” primes (Pennycook et al., 2021b; Pennycook et al., 2020), introducing friction (Fazio, 2020), and inoculation messages (Lewandowsky & van der Linden, 2021). Crucially, however, no theoretical framework exists for a nuanced evaluation of misinformation susceptibility, nor a psychometrically validated measurement that provides a reliable measure across studies.

## Inconsistent interpretation and the need for a new measurement instrument

Despite the plethora of research papers on the psychology of misinformation, the field has not converged on a standardized way of defining or measuring people’s susceptibility to misinformation. In the absence of such a commonly agreed-upon standard, scholars have been inventive in the way that they employ individually constructed misinformation tests, often with the best intentions to create a good scale, but typically without formal validation (e.g., Pennycook, Epstein, et al., 2021b; Roozenbeek et al., 2021b).

The extent of the problem becomes evident when examining how researchers develop their test items and report the success of their models or interventions. Typically, researchers create (based on commonly used misinformation techniques; e.g., Maertens et al., 2021; Roozenbeek & van der Linden, 2019) or select (from a reliable fact-check database; e.g., Cook et al., 2017; Guess et al., 2020; Pennycook et al., 2020; Pennycook & Rand, 2019; Swire et al., 2017; van der Linden et al., 2017) news headlines or social media posts, where participants rate the reliability, intentions to share, accuracy, or manipulativeness of these items on a Likert or binary (e.g., true vs. false) scale; for an extensive discussion, see Roozenbeek et al. (2022). Sometimes the news items are presented as plain-text statements (e.g., Roozenbeek et al., 2020), while in other studies researchers present headlines together with an image, source, and lede sentence (e.g., Pennycook & Rand, 2019). The true-to-false ratio often differs, where in some studies only false news items are presented (e.g., Roozenbeek et al., 2020), and in others this is an unbalanced (e.g., Roozenbeek et al., 2021b) or balanced (e.g., Pennycook & Rand, 2019) ratio of true and false items. Often an index score is created by taking the average of all item ratings (an index score reflecting general belief in false or true news items; e.g., Maertens et al., 2021), or by calculating the difference between ratings of true items and false items (veracity discernment; e.g., Pennycook, McPhetres, et al., 2020). Finally, an effect size is calculated, and a claim is made with respect to the effectiveness of the intervention, based on a change in false news ratings (e.g., Roozenbeek & van der Linden, 2019), a combined change in true news ratings and false news ratings (e.g., Guess et al., 2020), or even a change in true news ratings only (Pennycook, McPhetres et al., 2020).

It becomes clear that the wide variation in methodologies makes it hard to compare studies or generalize conclusions beyond the studies themselves. Little is known about the psychometric properties of these ad hoc scales and whether or not they measure a latent trait. As a widespread practice in misinformation research, scholars often assume—rather than know—that they are measuring the same construct. As a result, if this bold assumption turned out to be untrue, we would be at risk of obscuring underlying phenomena by incorrectly labeling them as the same mechanism, thereby engaging in an illusory essence bias (Brick et al., 2022) and/or falling prey to jingle fallacies (Block, 1995; Condon et al., 2020). As misinformation is a complex issue, the responses on one item set may be a result of motivational factors, while responses on another scale may be more reflective of critical thinking skills, instead of both measuring the same “discernment skill.” We currently do not know how different misinformation susceptibility scales are related, or how the true-to-false ratios influence their outcome (Aird et al., 2018) and how much of the effects found are due to response biases rather than changes in skill (Batailler et al., 2022). The limited studies that do look at the issue of scale-specific effects show significant item effects, indicating a risk of skewed conclusions about intervention effect sizes (e.g., Roozenbeek, Maertens et al., 2021b).^2^ Relatedly, whether the sampling of test items, their presentation, and response modes have a high ecological validity is often not discussed (Dhami et al., 2004; Roozenbeek et al., 2022), and little is known about the nomological net and reliability of the indices used. In other words, it is difficult to disentangle whether differences between studies are due to differences in the interpretation schema, the measurement instrument, or actual differences in misinformation susceptibility. This indicates a clear need for a unified theoretical framework in conjunction with a standardized instrument with strong internal and external validity.

## The present research

### Towards a universal conceptualization and measurement: The Verification done framework

**Fig. 1:**
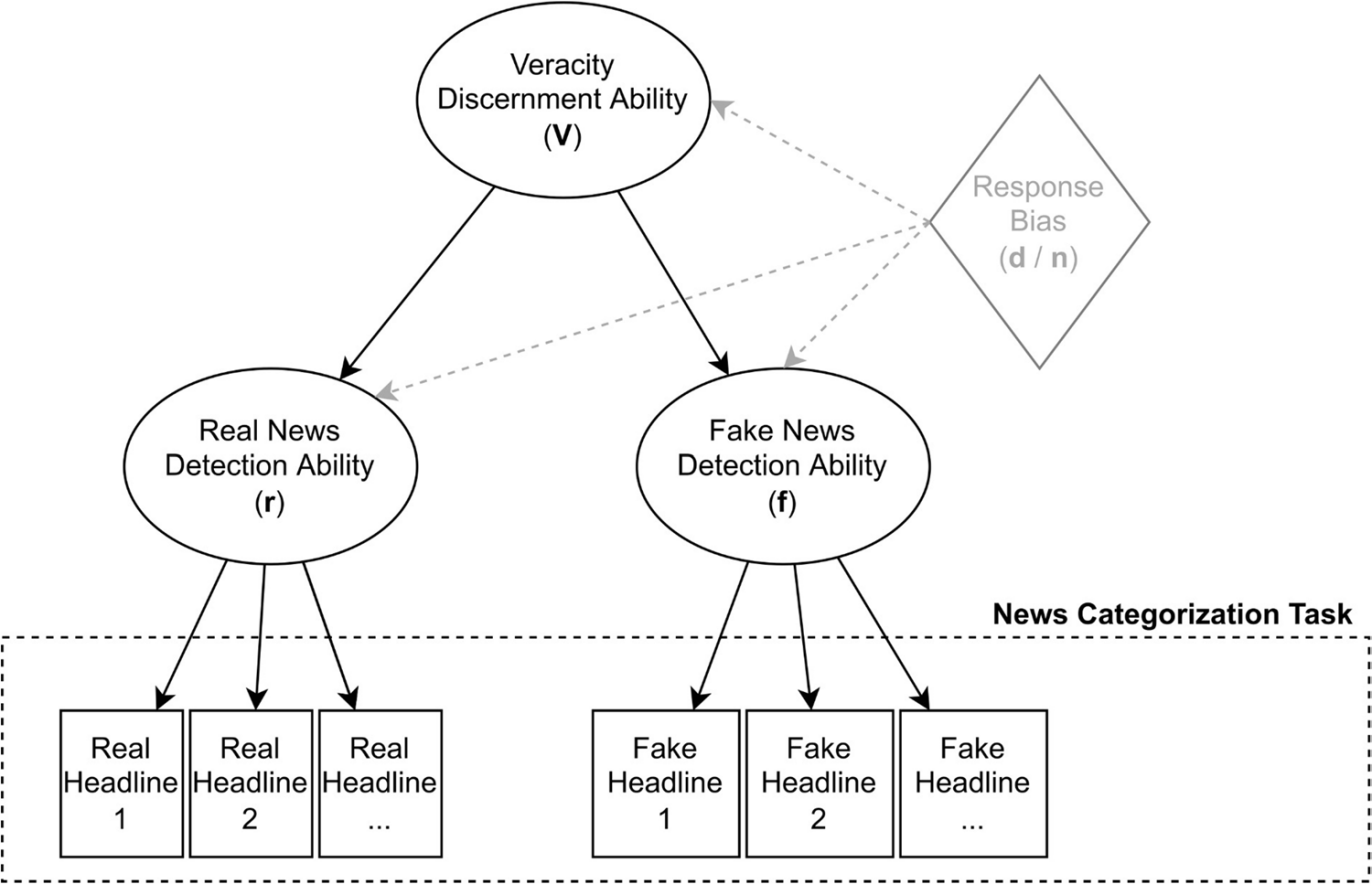
Visualization of the **V**e**r**i**f**ication **d**o**n**e model

Here, we set out to create a theoretical interpretation schema as well as a first psychometrically validated measurement instrument that, in conjunction, resolve the issues mentioned above and offer utility for a wide range of scholars. We extend the current literature by providing the first psychometrically integrated conceptualization of misinformation susceptibility that allows for a reliable *holistic* measurement through the ***V****e****r****i****f****ication* ***d****o****n****e* framework: we can only fully interpret misinformation susceptibility—or the impact of an intervention—by capturing *news veracity discernment* (**V**, ability to accurately distinguish real news from fake news) as a general factor, the specific facets *real news detection ability* (**r**, ability to correctly identify real news) and *fake news detection ability* (**f**, ability to correctly identify fake news), *distrust* (**d**; negative judgment bias, being overly skeptical), and *naïvité* (**n**; positive judgment bias, being overly gullible), and comparing **V**, **r**, **f**, **d**, *and* **n** alongside each other. A visualization of the ***V****e****r****i****f****ication* ***d****o****n****e* model can be found in Fig. 1. For example, two different interventions may increase discernment ability **V** to a similar extent, but intervention A might do so by increasing detection ability **r**, while intervention B may accomplish the same by increasing detection ability **f**. Similarly, two people with the same discernment ability **V** may have opposite **r** and **f** abilities. Changes in detection abilities **r** or **f** after an intervention have to be interpreted together with changes in judgment biases **d** and **n** to determine whether the intervention has done more than just increase a judgment bias. Existing interventions often look at a limited subset of these five dimensions; for example, the creators of the *Bad News Game* intervention (Roozenbeek & van der Linden, 2019) originally focused on *fake news detection*, including only a few real news items. Meanwhile, the *accuracy nudge* intervention seems to work mainly by addressing *real news detection* (Pennycook, McPhetres, et al. 2020), although we are not sure about the judgment biases. Another media literacy intervention was found to increase general distrust, but showed improvement on *veracity discernment* nevertheless (Guess et al., 2020).

In order to be able to compare these scores and gain insights into the complete picture, we need to employ the ***V****e****r****i****f****ication* ***d****o****n****e* framework, but also make sure that each scale has high validity and comparability. To accomplish this, through a series of three studies and using a novel neural-network-based item generation approach, we develop the Misinformation Susceptibility Test (MIST): a psychometrically validated (based on classical test theory and item response theory, as well as exploratory graph analysis) measurement instrument. The MIST was developed to be the first truly balanced misinformation susceptibility measure with an equal emphasis on discernment, real news detection, fake news detection, and judgment bias. In addition, to put the results into perspectives, all scores should be interpreted along with national norm tables. In the present study, we describe how we developed and validated the MIST to accomplish these goals, evaluate each of these dimensions, and investigate the practical utility of the MIST for researchers and practitioners in the field.

### The Misinformation Susceptibility Test

We conduct three studies to develop, validate, and apply the MIST. In Study 1 (*N* = 409), we employ a multitude of exploratory factor analysis (EFA)- and item response theory (IRT)-based selection criteria to create a 20-item MIST full-scale and an 8-item MIST short-scale from a larger item pool that was built using a combination of advanced language-based neural network algorithms and real news headline extraction from reliable and unbiased media outlets, and then pre-filtered through multiple iterations of expert review. The resultant MIST scales are balanced (50% real, 50% fake), binary (real/fake), cumulatively scored instruments that ask participants to rate presented news headlines as either true or false, with higher MIST scores indicating greater discernment ability.^3^ We also present a new, alternative method to EFA and IRT, namely exploratory graph analysis (EGA; Golino & Epskamp, 2017; Golino et al., 2021), to show how modern psychometrics may lead to other robust item selections.

We acknowledge that the typical news consumption diet in real life includes more real news than fake news (e.g., Guess et al., 2020). However, as misinformation has the potential to spread faster (Brady et al., 2017; Vosoughi et al., 2018), and we aim to accurately measure a *general* discernment ability as well as both real news detection and fake news detection, in creating the MIST we have given equal representation on both facets. This allows us to generalize across the board—independent of an individual’s news consumption ratio. Meanwhile, to capture any biases related to overly positive or negative responses (to news in general), we have later added a method to calculate response biases **d** and **n** (these were not part of the original scale development protocol). As such, the MIST exhibits a psychometrically validated higher-order structure, with two validated first-order factors **r** and **f** (i.e., real news detection, fake news detection) and one general ability second-order factor **V** (i.e., veracity discernment), as well as a method to calculate response biases **d** (i.e., distrust) and **n** (i.e., naïvité).^4^

In Study 2 (*N* = 7674), we employ confirmatory factor analyses (CFA), as well as EGA, to replicate the MIST’s structure across four national quota samples from the UK and the US, establish construct validity via a large, preregistered nomological network, and derive norm tables for the general populations of the UK and US and demographic and geographical subgroups.

In Study 3 (*N* = 421), we provide an example of how to implement ***V****e****r****i****f****ication* ***d****o****n****e* and the MIST in the field by applying it in the naturalistic setting of a well-replicated media literacy intervention, the *Bad News Game* (https://www.getbadnews.com/). Whereas ample prior studies have attested to the theoretical mechanisms and effects that contribute to the *Bad News Game*’s effectiveness in reducing misinformation susceptibility (see, e.g., Maertens et al., 2021; Roozenbeek & van der Linden, 2019), within-subject repeated-measures analyses of the MIST-8 for pre-and post-game tests in conjunction with the ***V****e****r****i****f****ication* ***d****o****n****e* framework reveal important new insights about how the intervention affects people across different evaluative dimensions. This paper demonstrates the benefits of integrated theory and assessment development, resulting in a framework providing nuanced, multifaceted insights that can be gained from a short, versatile, psychometrically sound, and easy-to-administer new measure. Table 1 offers a comprehensive summary of all samples used, detailing their size, demographic breakdowns, included measures, country of origin, recruitment platform, and whether or not they (a) used nationally representative quota and (b) were preregistered.

**Table 1:** Summary of samples

	Study 1: Development	Study 2: Validation					Study 3: Application
Sample	1A	2A	2B	2C	2D	2E	3
*N*	409	3479	510	1227	1245	1213	421
Country of origin	USA	USA	USA	UK	UK	USA	USA
Nationally representative quota	No	Yes	Yes	Yes	Yes	Yes	No
Recruitment platform	Prolific	Respondi	CloudResearch	Respondi	Prolific	Respondi	*Bad News Game*
Preregistration	Yes	No	Yes	No	No	No	No
Demographic composition	**Age** *M*_age_ = 33.20 *SD*_age_ = 11.85	**Age** *M*_age_ = 45.10 *SD*_age_ = 16.16	**Age** *M*_age_ = 49.25 *SD*_age_ = 16.96	**Age** *M*_age_ = 45.34 *SD*_age_ = 16.52	**Age** *M*_age_ = 44.66 *SD*_age_ = 15.65	**Age** *M*_age_ = 45.21 *SD*_age_ = 17.35	**Age** 55.58% [18, 29] 32.30% [30, 49] 12.11% [50, 99]
	**Gender** 55.50% female 42.30% male 2.20% other/nonbinary	**Gender** 51.11% female 48.84% male 0.06% other/nonbinary	**Gender** 55.88% female 43.53% male 0.59% other/nonbinary	**Gender** 51.67% female 48.33% male 0.00% other/nonbinary	**Gender** 52.53% female 47.07% male 0.40% other/nonbinary	**Gender** 54.00% female 44.19% male 1.81% nonbinary	**Gender** 52.02% female 41.09% male 6.89% other/nonbinary
	**Ethnicity** –	**Ethnicity** 76.89% White, Caucasian, Anglo, or European American 8.39% Asian or Asian American 6.00% Hispanic or Latino 5.98% Black or African American 1.12% Native American or Alaskan Native 0.54% Middle Eastern 0.30% Hawaiian or Pacific Islander 0.77% Other/Prefer not to answer	**Ethnicity** 68.81% White, Caucasian, Anglo, or European American 4.28% Asian or Asian American 11.05% Hispanic or Latino 12.12% Black or African American 2.50% Native American or Alaskan Native 0.18% Middle Eastern 1.07% Other/Prefer not to answer	**Ethnicity** 87.33% White 6.95% Asian 2.45% Black 0.08% Arab 2.13% Mixed 1.06% Other	**Ethnicity** 86.10% White 7.47% Asian 3.53% Black 0.16% Arab 1.61% Mixed 1.12% Other	**Ethnicity** –	**Ethnicity** –
	**Education** 1.47% Less than high school degree 9.29% High school graduate 31.30% Some college but no degree 38.88% Bachelor's degree in college 1.96% Professional degree 13.45% Master's degree 3.67% Doctoral degree	**Education** 1.74% Did not complete high school 34.98% High school degree or equivalent 15.08% Associate degree 31.84% Degree (bachelor’s) or equivalent 15.11% Degree (master’s) or other postgraduate qualification 1.25% Doctorate 0.97% Other/Prefer not to say	**Education** 2.55% Less than high school degree 25.10% High school graduate 27.45% Some college but no degree 26.08% Bachelor's degree in college 1.57% Professional degree 13.92% Master's degree 3.33% Doctoral degree	**Education** 11.03% No formal education above age 16 16.18% Professional or technical qualifications above age 16 27.12% School education up to age 18 31.94% Degree (bachelor’s) or equivalent 12.09% Degree (master’s) or other postgraduate qualification 1.63% Doctorate	**Education** 6.27% No formal education above age 16 10.68% Professional or technical qualifications above age 16 25.22% School education up to age 18 38.63% Degree (bachelor’s) or equivalent 16.87% Degree (master’s) or other postgraduate qualification 2.33% Doctorate	**Education** 2.72% Less than high school degree 24.73% High school graduate or equivalent 19.79% Some college, but no degree 11.54% Associate degree in college, 2-year 25.72% Bachelor’s degree in college, 4-year 12.45% Master’s degree 2.14% Professional degree, JD, MD 0.91% Doctoral degree	**Education** 14.49% High school or less 36.10% Some college 49.41% Higher degree
Measured constructs	*- MIST-100* *- BSR* *- CMQ* *- COVID-19 compliance* *- CRT* *- DEPICT* *- CV19 fact-check*	*- MIST-20 (incl. MIST-8)* *- AOT* *- Anti-vaccination attitudes* *- COVID-19 misinformation beliefs* *-CRT* *- Numeracy* *- Political ideology* *- Trust (in scientists, journalists, politicians, the government)*	*- MIST-20 (incl. MIST-8)* *- BSR* *- BFI2-S* *- CMQ* *- EDO* *- DEPICT SF* *- Go Viral!* *- MFQ20* *- SD4* *- SDO* *- SINS* *- SISES* *- SIRIS* *- SSPC* *- Trust (in medical personnel, scientists, politicians, journalists, the government, scientific knowledge, civil servants, mainstream media)*	*- MIST-20 (incl. MIST-8)* *- Numeracy* *- Political ideology* *- Trust (in medical personnel, scientists, politicians, journalists, the government, scientific knowledge, civil servants, mainstream media)*	*- MIST-20 (incl. MIST-8)* *- Numeracy* *- Political ideology* *- Trust (in medical personnel, scientists, politicians, journalists, the government, scientific knowledge, civil servants, mainstream media)*	*- MIST-16*	*- MIST-8* - *BN*

AOT = Actively Open-minded Thinking (Baron, 2019); BFI-2-S = Big-Five Inventory 2 Short-Form (Soto & John, 2017); BN = *Bad News Game* (Roozenbeek & van der Linden, 2019); BSR = Bullshit Receptivity scale (Pennycook et al., 2015); CMQ = Conspiracy Mentality Questionnaire (Bruder et al., 2013); CRT = Cognitive Reflection Test (Frederick, 2005); DEPICT = Discrediting-Emotion-Polarization-Impersonation-Conspiracy-Trolling deceptive headlines inventory (Maertens et al., 2021); DEPICT SF = DEPICT Balanced Short Form (Maertens et al., 2021); EDO = Ecological Dominance Orientation (Uenal et al., 2022); CV19 fact-check = COVID-19 fact-check task (Pennycook, McPhetres, et al., 2020); Go Viral! = Go Viral! Balanced Item Set (Basol et al., 2021); MFQ20 = Moral Foundations Questionnaire 20-Item Short Form (Graham et al., 2011); Numeracy = combination of Schwartz Numeracy Test (Schwartz et al., 1997) and Berlin Numeracy Test (Cokely et al., 2012), SD4 = Short Dark Tetrad (Paulhus et al., 2020); SDO = Social Dominance Orientation (Ho et al., 2015); SINS = the Single-Item Narcissism Scale (Konrath et al., 2014); SISES = Single-Item Self-Esteem Scale (Robins et al., 2001) SIRIS = Single-Item Religious Identification Scale (Norenzayan & Hansen, 2006); SSPC = Short Scale of Political Cynicism (Aichholzer & Kritzinger, 2016)

## Study 1: Development—Scale construction, exploratory analyses, and psychometric properties

**Fig. 2:**
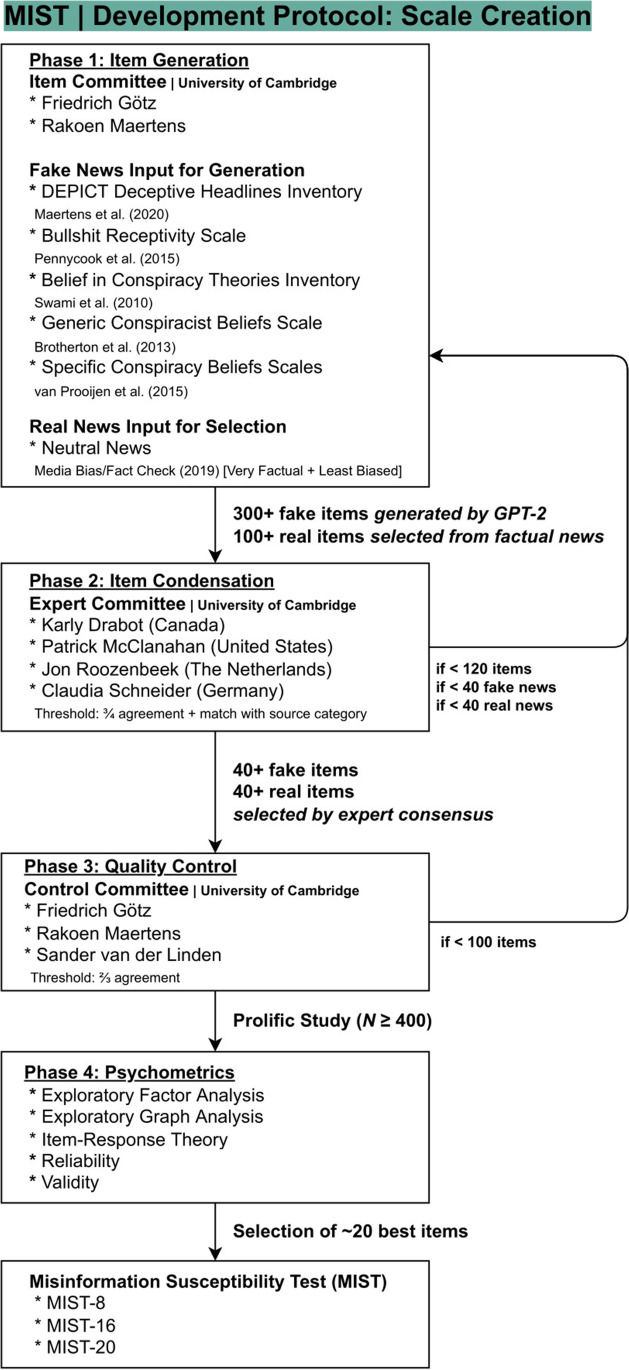
Development protocol of the Misinformation Susceptibility Test

Following classic (Clark & Watson, 1995; Loevinger, 1957) and recent (Boateng et al., 2018; Rosellini & Brown, 2021; Zickar, 2020) psychometrics guidelines, and taking into account insights from misinformation scholars (Pennycook et al., 2021a; Roozenbeek et al., 2021b), we devised a four-stage, preregistered scale development protocol (i.e., 1—item generation, 2—expert filtering, 3—quality control, and 4—data-driven selection), shown in Fig. 2.

## Method

### Preparatory steps

#### Phase 1: Item generation

##### Fake news

There is a debate in the literature on whether the misinformation items administered in misinformation studies should be actual news items circulating in society, or news items created by experts that are fictional but feature common misinformation techniques. The former approach arguably provides better ecological validity (Pennycook, Binnendyk, et al., 2021), while the latter provides a cleaner and less confounded measure since it is less influenced by memory and identity effects (van der Linden & Roozenbeek, 2020). Considering these two approaches and reflecting on representative stimulus sampling (Dhami et al., 2004), we opted for a novel approach that combines the best of both worlds. We employed the generative pretrained transformer 2 (GPT-2)—a neutral-network-based artificial intelligence developed by OpenAI (Radford et al., 2019)—to generate fake news items (cf., Götz et al., 2022; Hommel et al., 2022). The GPT-2 is one of the most powerful open-source text generation tools currently available for free use by researchers. It was trained on eight million text pages, combines 1.5 billion parameters, and is able to write coherent and credible articles based on just one or a few words of input.^5^ We did this by asking the GPT-2 to generate a list of fake news items inspired by a smaller set of items. This smaller set contained items from any of five different scales that encompass a wide range of misinformation properties: the Belief in Conspiracy Theories Inventory (BCTI; Swami et al., 2010), the Generic Conspiracist Beliefs scale (GCB; Brotherton et al., 2013), specific Conspiracy Beliefs scales (van Prooijen et al., 2015), the Bullshit Receptivity scale (BSR; Pennycook et al., 2015), and the Discrediting-Emotion-Polarization-Impersonation-Conspiracy-Trolling deceptive headlines inventory (DEPICT; Maertens et al., 2021; Roozenbeek & van der Linden, 2019). We set out to generate 100 items of good quality, but as this is a new approach, we opted for the generation of at least 300 items. More specifically, we let GPT-2 generate thousands of fake news headlines, and tossed out any duplicates and clearly irrelevant items (see Supplement S1 for a full overview of all items generated and those that have been removed).

##### Real news

For the real news items, we decided to include items that met each of the following three selection criteria: (1) the news items are actual news items (i.e., they circulated as real news), (2) the news source is the most factually correct (i.e., accurate), and (3) is the least biased (i.e., nonpartisan or politically centrist). To do this, we used the Media Bias/Fact Check database (MBFC; https://mediabiasfactcheck.com/) to select news sources marked as *least biased* and scoring *very high* on factual reporting.^6^ The news sources we chose were Pew Research (https://www.pewresearch.org/), Gallup (https://www.gallup.com/), MapLight (https://maplight.org/), Associated Press (https://www.ap.org/), and World Press Review (http://worldpress.org/). We also diversified the selection by including the non-US outlets Reuters (https://www.reuters.com/), Africa Check (https://africacheck.org/), and JStor Daily (https://daily.jstor.org/). All outlets received the maximum MBFC score at the time of item selection.^7^ A full list of the real news items selected can be found in Supplement S1.

Overall, this item-generation process resulted in an initial pool of 413 items. The full list of items we produced and methods through which each of them was obtained can be found in Supplement S1.

##### Phase 2: Item condensation

To reduce the number of headlines generated in Phase 1, we followed previous scale development research and practices (Carpenter, 2018; Haynes et al., 1995; Simms, 2008) and established an expert committee with misinformation researchers from four different cultural backgrounds: Canada, Germany, the Netherlands, and the United States. Each expert conducted an independent review and classified each of the 413 items generated in Phase 1 as either *fake news* or *real news*. All items with a three-fourths expert consensus *and* matching the correct answer key (i.e., the source veracity category)—a total of 289 items—were selected for the next phase.^8^ A full list of the expert judgments and inter-rater agreement can be found in Supplement S1.

##### Phase 3: Quality control

As a final quality control before continuing to the psychometrics study, the two-person item generation committee in combination with an extra third expert—who had not been previously exposed to any of the items—made a final selection of items from Phase 2. Applying a two-thirds expert consensus as cutoff, we selected 100 items (44 fake news, 56 real news) out of the 289 from the previous stage (i.e., we cut 189 items), thus creating a fairly balanced item pool for empirical probing that hosted five times as many items as the final scale that we aimed to construct—in keeping with conservative guidelines (Boateng et al., 2018; Weiner et al., 2012). A full list of the item sets selected per expert and expert agreement can be found in Supplement S1.

#### Implementation

##### Participants

In line with widespread recommendations to assess at least 300 respondents during initial scale implementation (Boateng et al., 2018; Clark & Watson, 1995, 2019; Comrey & Lee, 1992; Guadagnoli & Velicer, 1988), we recruited a community sample of 452 US residents (for a comprehensive sample description see Table 1). The study was carried out on Prolific Academic (https://www.prolific.co/), an established crowd-working platform which provides competitive data quality (Palan & Schitter, 2018; Peer et al., 2017). Based on the exclusion criteria laid out in the preregistration, we removed incomplete cases, participants who took either an unreasonably short or long time to complete the study (less than 8 minutes or more than 2 hours), participants who failed an attention check, underage participants, and participants who did not live in the United States, retaining 409 cases for data analysis.^9^ Of these, 225 participants (i.e., 55.01%) participated in the follow-up data collection eight months later (T2).^10^

Participants received a set remuneration of 1.67 GBP (equivalent to US$ 2.32) for participating in the T1 questionnaire and 1.10 GBP (equivalent to US$ 1.53) for T2.

#### Procedure, measures, transparency, and openness

The preregistrations for T1 and T2 are available on AsPredicted https://aspredicted.org/m7vb3.pdf; https://aspredicted.org/js2jz.pdf; any deviations can be found in Supplement S2). The supplement, raw and clean datasets, and all analysis scripts in R can be found in the OSF repository (https://osf.io/r7phc/).

Participants took part in a preregistered online survey. After providing informed consent, participants had to categorize the 100 news headlines from Phase 3 (i.e., the items that were retained after the previous three phases) in two categories: *Fake/Deceptive* and *Real/Factual*.^11^ Participants were told that each headline had only one correct answer. See the preregistration or the Qualtrics files on the OSF repository for the exact survey framing (https://osf.io/r7phc/).

After completing the 100-item categorization task, participants completed the 21 items from the DEPICT inventory (a misleading social media post reliability judgment task; Maertens et al., 2021), a 30-item COVID-19 fact-check task (a classical true/false headline evaluation task; Pennycook, McPhetres, et al., 2020), the Bullshit Receptivity scale (BSR; Pennycook et al., 2015), the Conspiracy Mentality Questionnaire (CMQ; Bruder et al., 2013), the Cognitive Reflection Test (CRT; Frederick, 2005), a COVID-19 compliance index (sample item: “I kept a distance of at least two meters to other people”: 1 – *does not apply at all*, 4 – *applies very much*), and a demographics questionnaire (see Table 1 for an overview). Finally, participants were debriefed. Eight months later, the participants were recruited again for a test-retest follow-up survey.^12^ In the follow-up survey, after participants provided informed consent to participate, the final 20-item MIST was administered, the same COVID-19 fact-check task (Pennycook, McPhetres, et al., 2020) and CMQ (Bruder et al., 2013) were repeated, a new COVID-19 compliance index was administered, and finally a full debrief was presented. The complete surveys are available in the OSF repository: https://osf.io/r7phc/.

The full study received institutional review board (IRB) approval from the Psychology Research Ethics Committee of the University of Cambridge (PRE.2019.108).

#### Analytical strategy 1: Exploratory factor analysis (EFA) and item response theory (IRT)

To extract the final MIST-20 and MIST-8 scales from the pre-filtered MIST-100 item pool, we followed an item selection decision tree, which can be found in Supplement S3. Specifically—after ascertaining the general suitability of the data for such procedures—the following EFA- and IRT-based exclusion criteria were employed: (1) factor loadings below .40 (Clark & Watson, 2019; Ford et al., 1986; Hair et al., 2010; Rosellini & Brown, 2021); (2) cross-loadings above .30 (Boateng et al., 2018; Costello & Osborne, 2005); (3) communalities below .4 (Carpenter, 2018; Fabrigar et al., 1999; Worthington & Whittaker, 2006); (4) Cronbach’s α reliability analysis; (5) differential item functioning (DIF) analysis (Holland & Wainer, 1993; Nguyen et al., 2014; Reise et al., 1993); (6) item information function (IIF) analysis. Finally, we sought to establish initial evidence for construct validity (Cronbach & Meehl, 1955). To do this, we investigated the associations between the MIST scales and the DEPICT deceptive headline recognition task (Maertens et al., 2021) and COVID-19 fact-check (Pennycook et al., 2020; concurrent validity). We further examined additional predictive accuracy of the MIST in accounting for variance in DEPICT and fact-check scores above and beyond the CMQ (Bruder et al., 2013), BSR (Pennycook et al., 2015), and CRT (Frederick, 2005; incremental validity).

#### Analytical strategy 2: Exploratory graph analysis (EGA)

In this section we explore an alternative method of scale development, based on the new field of exploratory graph analysis (Golino & Epskamp, 2017), rooted in network methods. Network methods in psychology gained momentum with the publication of the mutualism model of intelligence (Van Der Maas et al., 2006) and network perspective on psychopathology (Borsboom, 2008; Borsboom et al., 2011; Cramer et al., 2010), giving rise to a new subfield of quantitative psychology called *network psychometrics* (Epskamp et al., 2017; Epskamp et al., 2018). Network models are used to estimate the relationship between multiple variables—typically using the Gaussian graphical model (GGM; Lauritzen, 1996), where *nodes* (e.g., test items) are connected by *edges* (or links) that indicate the strength of the association between the variables (Epskamp & Fried, 2018), forming a system of mutually reinforcing elements (Christensen et al., 2020b; Cramer, 2012). Network and latent variable models have been shown to be closely related, and can produce model parameters that are consistent with one another (Boker, 2018; Christensen & Golino, 2021c; Epskamp et al., 2017; Golino et al., 2021; Golino & Epskamp, 2017; Marsman et al., 2018). These statistical similarities can be used as a way to explore the dimensionality structure of measurement instruments in a new framework termed *exploratory graph analysis* (Christensen et al., 2019; Golino & Demetriou, 2017; Golino & Epskamp, 2017; Golino et al., 2020a, 2020b).

In *network psychometrics* (Christensen et al., 2019; Epskamp et al., 2018; Epskamp et al., 2017; Golino & Demetriou, 2017; Golino & Epskamp, 2017; Golino et al., 2020a, 2020b), networks are typically estimated using the Gaussian graphical model (Lauritzen, 1996) using the *EBICglasso* approach (Epskamp & Fried, 2018). The *EBICglasso* approach operates by minimizing a penalized log-likelihood function and selecting the best model fit (i.e., the optimum level of sparsity in a network) using the extended Bayesian information criterion (EBIC; Chen & Chen, 2008). As Golino et al. (2022) argue, the use of weighted network models in psychology opened the doors for network science methods developed in other areas of science to psychological problems such as dimensionality (e.g., factor analysis).

Exploratory graph analysis was originally proposed by Golino and Epskamp et al. (2017), which showed that the GGM model combined with a clustering algorithm for weighted networks (*Walktrap*; Pons & Latapy, 2005) could accurately recover the number of simulated factors, presenting higher accuracy than traditional factor analytic-based methods. Later, Golino, Shi, et al. (2020b) compared EGA with different types of factor analytic methods (including two types of parallel analysis), finding that EGA achieves the highest overall accuracy (87.91%) in estimating the number of simulated factors, followed by the traditional parallel analysis with principal components of Horn (1965; 83.01%), and parallel analysis using principal axis factoring proposed by Humphreys and Ilgen (1969; 81.88%).

Golino et al. (2022) summarized the advantages of the EGA framework over more traditional methods (Golino, Shi, et al., 2020b): (1) unlike exploratory factor analysis (EFA) methods, EGA does not require a rotation method to interpret the estimated first-order factors (although rotations are rarely discussed in the validation literature, they have significant consequences for validation, e.g., estimation of factor loadings; Sass & Schmitt, 2010); (2) EGA automatically places items into factors without the researcher’s direction, which contrasts with exploratory factor analysis, where researchers must decipher a factor loading matrix (such a placement opens the door for dimension and item stability methods, which is presented next); and (3) the network representation depicts how items relate within and between dimensions.

Over the past couple of years, the EGA framework has expanded into several important areas of psychometrics. Christensen and Christensen and Golino (2021c) developed a new metric termed *network loadings* computed by standardizing node strength—the sum of the edges a node is connected to—split between dimensions identified by EGA. Christensen and Christensen and Golino (2021c) showed in their simulation study that network loadings are akin to factor loadings, but with different reference values. Network loadings of .15, .25, and .35 are equivalent to low (.40), moderate (.55), and high (.70) network loadings, respectively (Christensen & Golino, 2021c). The development of network loadings opened new lines of research, such as the development of metric invariance using EGA and permutation tests in a network perspective (Jamison et al., 2022), and determining whether data are generated from a factor or network model (Christensen & Golino, 2021b).

Based on the automated item placement of EGA, Christensen and Golino (2021a) developed a bootstrap approach to investigate the stability of items and dimensions estimated by EGA, termed *bootstrap exploratory graph analysis*, and proposed two new metrics of psychometric quality: *item stability* and *structural consistency*. Item stability indicates how often an item replicates in their designated EGA dimension, with values lower than .75 (i.e., that are estimated in their original dimensions in 75% of the bootstrapped samples) indicating problematic (or unstable) items. Structural consistency, by its turn, indicates how often an EGA dimension exactly replicates and can be used to verify configural (or structural) invariance and determine poor-functioning items (Golino et al., 2022). A complementary approach, called *unique variable analysis*, was developed to identify redundant items and can be used to identify the reason why some items function poorly (Christensen, Garrido, & Golino, 2020a).

The fit of a dimensionality structure estimated using EGA to the data can be verified using an innovative fit index termed *total entropy fit index* (*TEFI*; Golino, Moulder, et al., 2020a), developed as an alternative to traditional fit measures used in factor analysis and structural equation modeling (SEM). In a comprehensive simulation study, the *TEFI* demonstrated higher accuracy in correctly identifying the number of simulated factors than the comparative fit index (*CFI*), the root mean square error of approximation (*RMSEA*), and other indices used in SEM (Golino, Moulder, et al., 2020a). The *TEFI* is based on the Von Neumann entropy (Von Neumann, 1927)—a measure developed to quantify both the amount of disorder in a system and the entanglement between two subsystems (Preskill, 2018). The *TEFI* index is a relative measure of fit that can be used to compare two or more dimensionality structures. The dimensionality structure with the lowest *TEFI* value indicates the best fit for the data.

Another recent development within the EGA framework is the hierarchical EGA (*hierEGA*) technique by Jimenez et al. (2022). In their work, Jimenez et al. (2022) proposed an alternative variation to a popular clustering algorithm called Louvain (Blondel et al., 2008) to detect lower- and higher-order factors in data, and showed that this new technique is more effective than traditional factor analytic techniques to estimate the structure of first- and second-order factors in generalized bifactor structures.

All the EGA-based techniques/metrics mentioned above use the free and open-source R package *EGAnet* (Golino & Christensen, 2019), which has become one of the main software programs in network psychometrics. In the current paper, version 1.2.4 of the *EGAnet* package (Golino & Christensen, 2019) was used, and several strategies were implemented. The first strategy aimed at estimating the dimensionality structure of the 100 MIST items. Then, redundant items were identified using *unique variable analysis* (Christensen et al., 2020a), and for every group or pair of redundant items the one with the higher ratio of main network loadings to cross-loadings was kept in the analysis. The stability of the items and the structural consistency of the dimensions were obtained via *bootstrap exploratory graph analysis* (Christensen & Golino, 2021a) with 500 iterations (using parametric bootstrapping), and items with stability lower than 75% and network loadings lower than .15 were removed from subsequent steps. Once a subset of stable items with at least low to moderate network loadings were found, a subset of the best items per dimension (i.e., with moderate to high network loadings—with a network loading of at least .23) were identified, and further item stability and structural consistency metrics were computed until all items were highly stable (with item stability greater than 90%). The metric invariance of the final pool of best items per dimension (moderate to high network loadings and high item stability) was investigated using the EGA permutation test developed by Jamison et al. (2022), having as reference groups sex, age (above or below the median birth year), and education (above or below the median level of formal education received). The fit of the EGA-estimated dimensions to the data was computed using the total entropy fit index (Golino, Moulder, et al., 2020a) and compared to the two-factor structure of real and fake news items identified using EFA. *CFI* and *RMSEA* computed after fitting a confirmatory factor model to the EGA-estimated dimensions were also obtained, and compared to the *CFI* and *RMSEA* of the two-factor structure. Additionally, the Satorra (Satorra, 2000) scaled difference test was implemented to verify the structure with the best fit to the data.

### Results

#### EFA/IRT results

##### Item selection

Using parallel analysis with the *psych* package (Revelle, 2021), we aimed to select a parsimonious factor structure, with each factor reflecting eigenvalues above the 95th percentile of corresponding eigenvalues from 500 simulated random datasets.^13^ Parallel analysis (with 500 iterations) suggested a total of six factors, but only five factors (eigenvalues: F_1_ = 10.89, F_2_ = 7.82, F_3_ = 1.89, F_4_ = 1.42, F_5_ = 1.23, F_6_ = 0.98) matched our criteria and were above the 95th percentile of corresponding eigenvalues from the 500 simulated random datasets (eigenvalue 95th percentile = 0.99).^14^ Two factors explained most of the variance, which is in line with our theoretical model of two main factors (fake news detection and real news detection). An EFA using the tetrachoric correlation matrix with *unweighted least squares* (ULS) estimation without rotation using the *EFAtools* package (Steiner & Grieder, 2020) indicated that for both the two-factor structure and the five-factor structure, the first two factors were specifically linked to the real news items and the fake news items, respectively, while the other three factors did not show a pattern easy to interpret and in general showed low factor loadings (< .30).^15^ See Supplement S4 for a pattern matrix.

As we set out to create a measurement instrument for two distinct abilities, real news detection and fake news detection, we continued with a two-factor EFA, employing principal axis factoring and varimax rotation using the *psych* package (Revelle, 2021).^16^ Theoretically we would expect a balancing out of positive and negative correlations between the two factors: positive because of the underlying veracity discernment ability, and negative because of the response biases. In line with this, we chose an orthogonal rotation instead of an oblique rotation to separate out fake news detection and real news detection as cleanly as possible.

Three iterations were needed to remove all items with a factor loading under .40 (43 items were removed). After this pruning, no items showed cross-loadings larger than .30. Communality analysis using the three-parameter logistic model function in the *mirt* package (Chalmers, 2012) with 50% guessing chance (*c* = .50) indicated two items with communality lower than .40 after one iteration. These items were removed. No further iterations yielded any additional removals. A final list of the communalities can be found in Supplement S5. Cronbach’s α reliability analysis with the *psych* package was used to remove all items that had negative effects (∆α > .001) on the overall reliability of the test (Revelle, 2021). No items had to be removed based on this analysis.^17^ Differential item functioning using the *mirt* package was used to explore whether differences in gender or ideology would alter the functioning of the items (Chalmers, 2012). None of the items showed differential functioning for gender or ideology.

**Fig. 3:**
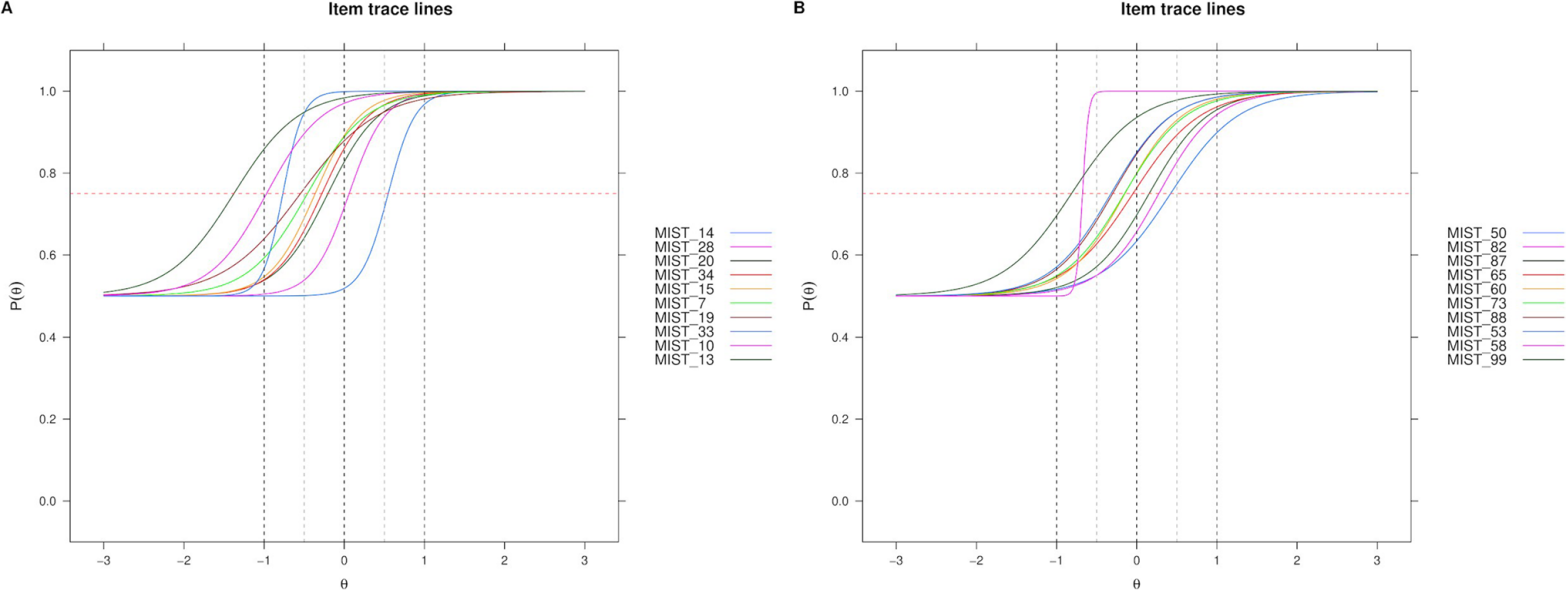
Item trace lines for MIST-20 items, for the fake news items in Panel **A** and real news items in Panel **B**. The items in the legend are ordered according to their difficulty level

**Fig. 4:**
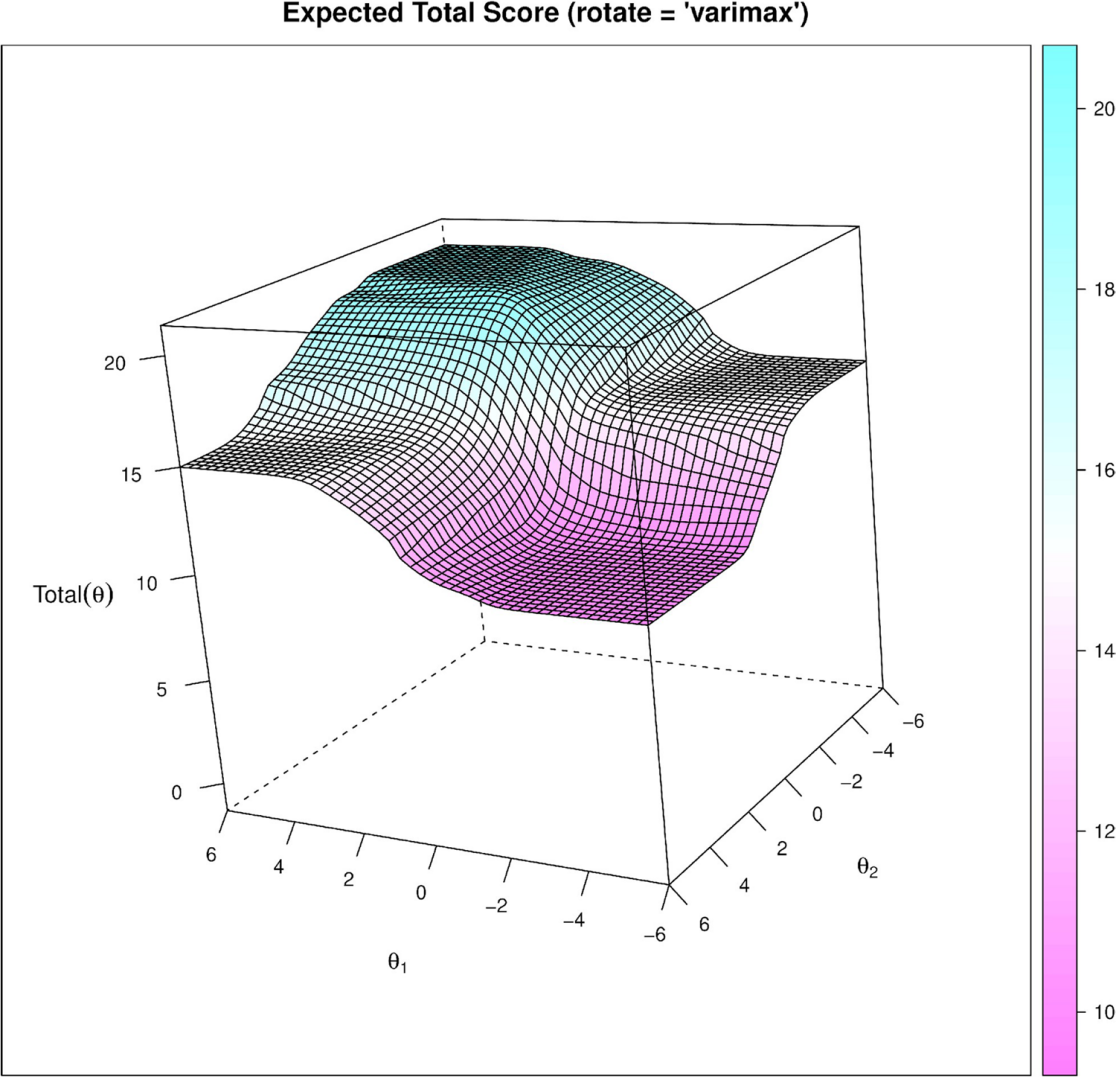
Multidimensional IRT plot representing the final MIST-20 test

Finally, using the three-parameter logistic model IRT functions in the *mirt* package (Chalmers, 2012), we selected the 20 best items (10 fake, 10 real) and the 8 best items (4 fake, 4 real), resulting in the MIST-20 and the MIST-8, respectively. These items were selected based on their discrimination and difficulty values, where we aimed to select a diverse set of items that have high discrimination (*a* ≥ 2.00 for the MIST-20, *a* ≥ 3.00 for the MIST-8) yet have a wide range of difficulties (*b* = [−0.50, 0.50], for each ability), while keeping the guessing parameter at 50% chance (*c* =.50). We also took into account the topics to ensure both that we covered a wide range of news areas *and* that there was no repetition of content (Flake et al., 2017). A list of the IRT coefficients and plots can be found in Supplement S1 and Supplement S6, respectively. See Fig. 3 for a MIST-20 item trace line plot, and Fig. 4 for a multidimensional plot of the MIST-20 IRT model predictions. The final items that make up the MIST-20 and MIST-8 are shown in Table 2.^18^ An overview of different candidate sets and how they performed, as well as the full analysis scripts and the supplement, can be found in the OSF repository: https://osf.io/r7phc/.

**Table 2:** Final items selected for MIST-20 and MIST-8

Item no.	*a*	*b*	Content
*Fake news*			
**MIST_14**	**3.50**	**0.53**	**Government Officials Have Manipulated Stock Prices to Hide Scandals**
MIST_28	2.69	0.06	The Corporate Media Is Controlled by the Military-industrial Complex: The Major Oil Companies Own the Media and Control Their Agenda
**MIST_20**	**3.26**	**−0.20**	**New Study: Left-Wingers Are More Likely to Lie to Get a Higher Salary**
MIST_34	3.42	−0.25	The Government Is Manipulating the Public's Perception of Genetic Engineering in Order to Make People More Accepting of Such Techniques
MIST_15	2.34	−0.40	Left-Wing Extremism Causes 'More Damage' to World Than Terrorism, Says UN Report
**MIST_7**	**2.57**	**−0.45**	**Certain Vaccines Are Loaded with Dangerous Chemicals and Toxins**
MIST_19	2.00	−0.55	New Study: Clear Relationship Between Eye Color and Intelligence
**MIST_33**	**5.60**	**−0.76**	**The Government Is Knowingly Spreading Disease Through the Airwaves and Food Supply**
MIST_10	2.64	−1.02	Ebola Virus 'Caused by US Nuclear Weapons Testing', New Study Says
MIST_13	2.86	−1.30	Government Officials Have Illegally Manipulated the Weather to Cause Devastating Storms
*Real news*			
**MIST_50**	**3.12**	**0.38**	**Attitudes Toward EU Are Largely Positive, Both Within Europe and Outside It**
MIST_82	2.22	0.31	One-in-Three Worldwide Lack Confidence in NGOs
MIST_87	2.25	0.14	Reflecting a Demographic Shift, 109 US Counties Have Become Majority Nonwhite Since 2000
MIST_65	2.36	−0.03	International Relations Experts and US Public Agree: America Is Less Respected Globally
**MIST_60**	**3.39**	**−0.09**	**Hyatt Will Remove Small Bottles from Hotel Bathrooms by 2021**
MIST_73	2.43	−0.14	Morocco’s King Appoints Committee Chief to Fight Poverty and Inequality
**MIST_88**	**2.79**	**−0.31**	**Republicans Divided in Views of Trump’s Conduct, Democrats Are Broadly Critical**
MIST_53	2.12	−0.37	Democrats More Supportive than Republicans of Federal Spending for Scientific Research
**MIST_58**	**8.59**	**−0.60**	**Global Warming Age Gap: Younger Americans Most Worried**
MIST_99	2.26	−0.83	US Support for Legal Marijuana Steady in Past Year

Items in bold are items included in the short version of the test (MIST-8). *a* = discrimination parameter. *b* = difficulty parameter

##### Reliability

Inter-item correlations show good internal consistency for both the MIST-8 (*IIC*_min_ = .20, *IIC*_max_ = .27) and the MIST-20 (*IIC*_min_ = .22, *IIC*_max_ = .29). Item-total correlations also show good reliability for both the MIST-8 (*ITC*_min_ = .44, *ITC*_max_ = .53) and the MIST-20 (*ITC*_min_ = .31, *ITC*_max_ = .54).

Looking further into the MIST-20, we analyze the reliability of veracity discernment (**V**; *M* = 15.71, *SD* = 3.35), real news detection (**r**; *M* = 7.62, *SD* = 2.43), and fake news detection (**f**; *M* = 8.09, *SD* = 2.10). In line with the guidelines by Revelle and Condon (2019), we calculate a two-factor McDonald’s ω (McDonald, 1999) as a measure of internal consistency using the *psych* package (Revelle, 2021), and find good reliability for the general scale and the two facet scales (ω_g_ = 0.79, ω_F1_ = 0.78, ω_F2_ = 0.75). Also using the *psych* package (Revelle, 2021), we calculate the variance decomposition metrics as a measure of stability, finding that F1 explains 14% of the total variance and F2 explains 12% of the total variance. Of all variance explained, 53% comes from F1 (**r)** and 47% comes from F2 (**f**), demonstrating a good balance between the two factors.

Finally, test–retest reliability analysis indicates that MIST scores are moderately positively correlated over a period of eight to nine months (*r*_T1,T2_ = 0.58).^19^

##### Validity

To assess initial validity, we examined the associations between the MIST scales and two scales that have been used regularly in previous misinformation research—the COVID-19 fact-check by Pennycook, McPhetres, et al. (2020) and the DEPICT task by Maertens et al. (2021)—expecting high correlations (*r* > .50; concurrent validity) and *additional* variance explained as compared to the existing CMQ, BSR, and CRT scales (incremental validity; Clark & Watson, 2019; Meehl, 1978). As can be seen in Table 3, we found that the MIST-8 displays a medium to high correlation with the fact-check (*r*_fact-check,MIST-8_ = .49) and DEPICT task (*r*_DEPICT,MIST-8_ = .45), while the MIST-20 shows a large positive correlation with both the fact-check (*r*_fact-check,MIST-20_ = .58) and the DEPICT task (*r*_DEPICT,MIST-20_ = .50). Using a linear model, we found that the explained variance in the fact-check indicates that the MIST-20 can explain 33% (adjusted *R*^2^) of variance by itself. The CMQ, BSR, and CRT combined account for 19%. Adding the MIST-20 on top provides an incremental 18% of explained variance (adjusted *R*^2^ = 0.37). The MIST-20 is the strongest predictor in the combined model (*t*(404) = 10.82, *p* < .001, β = 0.49, 95% CI [0.40, 0.57]). For the DEPICT task we found that the CMQ, BSR, and CRT combined explain 12% of variance in deceptive headline recognition and 26% when the MIST-20 is added (∆*R*^2^ = 0.14), while the MIST-20 alone explains 25%. For the DEPICT task we found the MIST-20 to be the only significant predictor in the combined model (*t*(404) = 8.94, *p* < .001, β = 0.43, 95% CI [0.34, 0.53]).^20^

**Table 3:** Incremental validity of MIST-8 and MIST-20 with existing measures

	*r*	Adjusted *R*^2^	∆*R*^2^
CV19 fact-check ~			
MIST-8	.49	.24	
MIST-20	.58	.33	
-			
CMQ + BSR + CRT		.19	
CMQ + BSR + CRT + MIST-8		.30	.11***
-			
CMQ + BSR + CRT		.19	
CMQ + BSR + CRT + MIST-20		.37	.18***
DEPICT ~			
MIST-8	.45	.20	
MIST-20	.50	.25	
-			
CMQ + BSR + CRT		.12	
CMQ + BSR + CRT + MIST-8		.22	.11***
-			
CMQ + BSR + CRT		.12	
CMQ + BSR + CRT + MIST-20		.26	.14***

* *p* < .05, ** *p* < .01, *** *p* < .001

### EGA results

**Fig. 5:**
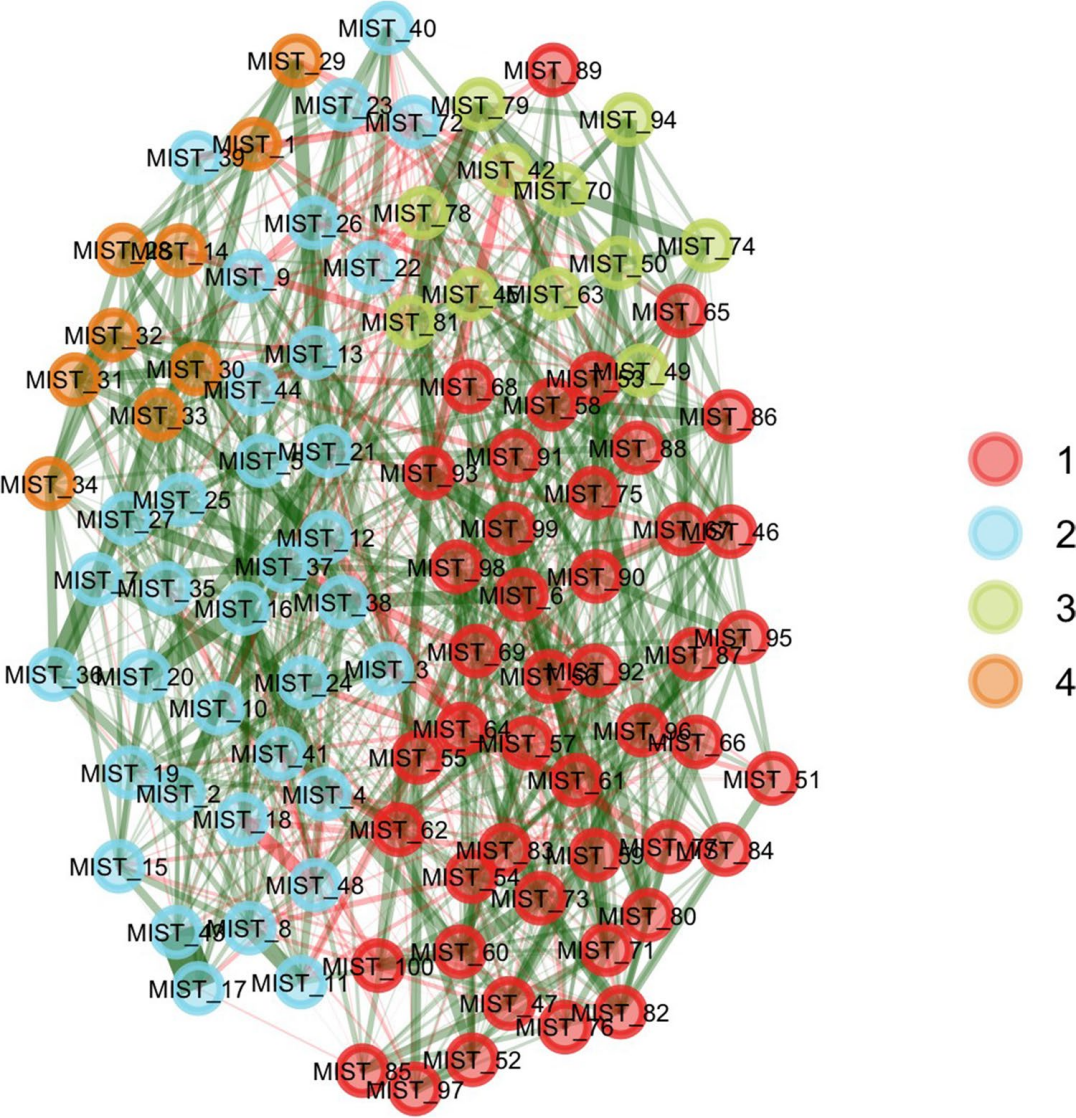
Structure of the 100 MIST items estimated using exploratory graph analysis

In this section we re-analyze the pool of 100 MIST items using EGA. EGA estimated four dimensions (see Fig. 5), which can be identified as two dimensions of real news headlines and two of fake news headlines. Dimension 1 (red nodes on Fig. 5) is a combination of US and international real news headlines, with items such as *MIST 96* (*US Hispanic Population Reached New High in 2018, But Growth Has Slowed*), *MIST 92* (*Taiwan Seeks to Join Fight Against Global Warming*), and *MIST 60* (*Hyatt Will Remove Small Bottles from Hotel Bathrooms by 2021*). Dimension 2 (blue nodes on Fig. 5) has fake news items about science, such as item *MIST 8* (*Climate Scientists’ Work Is “Unreliable”, a “Deceptive Method of Communication”*), and false statements against people with a liberal world view, such as items *MIST 16* (*Left-Wingers Are More Likely to Lie to Get a Good Grade*) and *MIST 20* (*New Study: Left-Wingers Are More Likely to Lie to Get a Higher Salary*). The third dimension (green nodes on Fig. 5) has real news items related to politically charged topics in the US, such as items *MIST 70* (*Majority in US Still Want Abortion Legal, with Limits*), *MIST 74* (*Most Americans Say It’s OK for Professional Athletes to Speak out Publicly about Politics*), and *MIST 94* (*United Nations Gets Mostly Positive Marks from People Around the World*). Dimension 4 (orange nodes on Fig. 5) has fake news items related to general conspiracy beliefs, such as item *MIST 1* (*A Small Group of People Control the World Economy by Manipulating the Price of Gold and Oil),* and conspiracies related to the government, such as items *MIST 31* (*The Government Is Actively Destroying Evidence Related to the JFK Assassination*) and *MIST 32* (*The Government Is Conducting a Massive Cover-Up of Their Involvement in 9/11*).

**Fig. 6:**
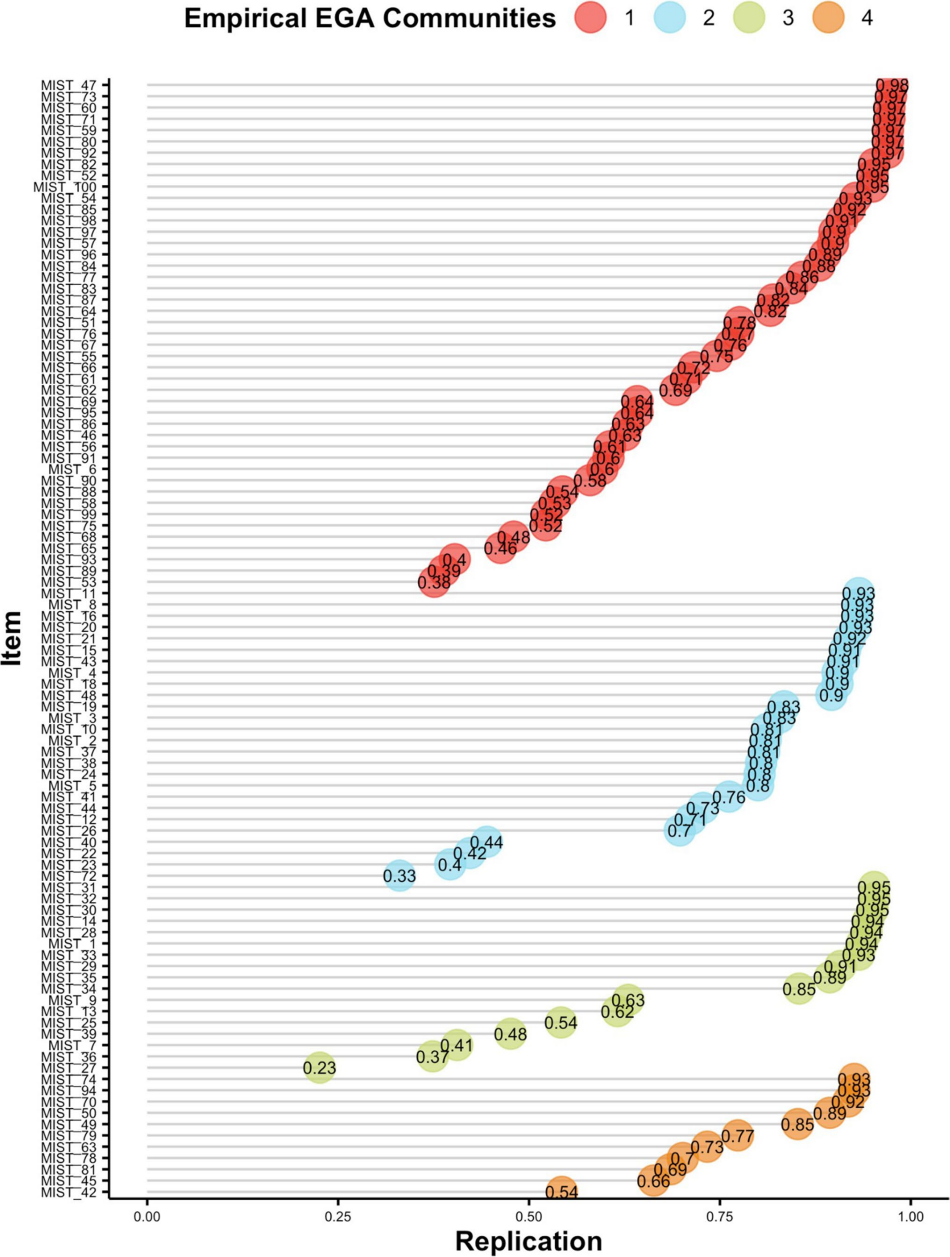
Item stability metric of the MIST-100 items in Study 1

The unique variable analysis technique (Christensen et al., 2020a) identified two redundant items: *MIST 43* (*UN: New Report Shows Shark Fin Soup as ‘the Most Important Source of Protein’ for World’s Poor*) and *MIST 17* (*New Data Show Shark Fins Are the ‘Most Important Source of Protein’ for the World’s Poor*). The ratio of network loadings (main/cross-loadings) for these items (8.47 and 6.9, respectively) suggested that item *MIST 43* should be kept in the subsequent analyses. A bootstrap exploratory graph analysis with 500 iterations (parametric bootstrapping) identified four median dimensions (95% CI: 2.11, 5.89) but with very low structural consistency for each dimension (0.09, 0.14, 0.07, and 0.43 for dimensions 1, 2, 3, and 4, respectively). The item stability metric (Christensen & Golino, 2021a) varied from 23% to 98%, with 40% of items presenting inadequate or moderate stability (i.e., lower than 75%, see Fig. 6).

Removing the items with item stability lower than 75% and repeating the parametric bootstrap EGA technique with 500 iterations showed that the stability improved considerably, leading to structural consistency between 0.61 (dimension 2) and 0.96 (dimension 4), and mean item stability of 93%. From the 59 items selected in the steps above, a subset with network loadings equal to or higher than .155 were selected from each dimension estimated via EGA, resulting in 34 items. A parametric bootstrap EGA with 500 iterations followed by item stability analysis was implemented once again, and items with stability lower than 75% were removed, resulting in 32 items.

**Fig. 7:**
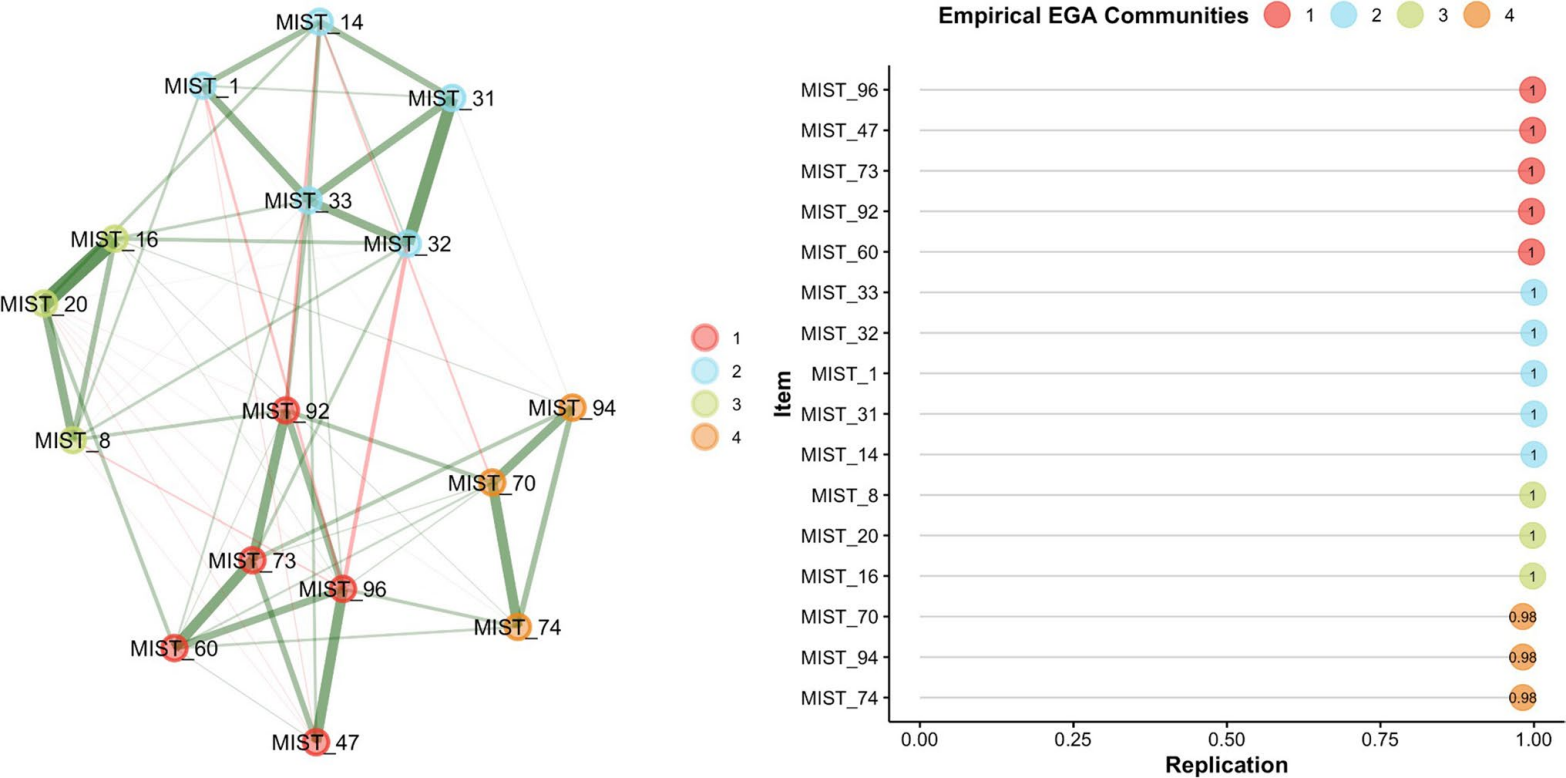
Final structure of the MIST-16 EGA items (left) and their stability indices (right) estimated using parametric bootstrap EGA with 500 iterations

The final selection of items was implemented using the following strategy. Out of the 32 items selected in the previous steps, only those with relatively high network loadings (≥ .23 or ≥ . 235) were used in the subsequent *bootEGA* and item stability analysis, which identified 16 highly stable items (see Fig. 7). Exploratory graph analysis identified the same four dimensions described in the first paragraph of this section, but now they presented very high structural consistency ranging from .982 to 1, and very high item stability (ranging from 98 to 100%). The network loadings of the final *MIST-16 EGA* items are presented in Table 4.

**Table 4:** Network loadings per item and dimension estimated via EGA. Network loadings of .15, .25, and .35 are equivalent to low (.40), moderate (.55), and high (.70) network loadings, respectively (Christensen & Golino, 2021c)

Item	Dim_1_	Dim_2_	Dim_3_	Dim _4_	Dim	Headline
MIST_73	0.35	0.04	−0.01	0.11	1	Morocco’s King Appoints Committee Chief to Fight Poverty and Inequality
MIST_96	0.33	−0.12	−0.06	0.10	1	US Hispanic Population Reached New High in 2018, But Growth Has Slowed
MIST_60	0.28	0.03	0.07	0.10	1	Hyatt Will Remove Small Bottles from Hotel Bathrooms by 2021
MIST_92	0.24	0.11	0.08	0.09	1	Taiwan Seeks to Join Fight Against Global Warming
MIST_47	0.24	0.06	−0.03	0.00	1	About a Quarter of Large US Newspapers Laid off Staff in 2018
MIST_33	0.16	0.40	0.06	0.00	2	The Government Is Knowingly Spreading Disease Through the Airwaves and Food Supply
MIST_31	0.00	0.40	0.00	0.01	2	The Government Is Actively Destroying Evidence Related to the JFK Assassination
MIST_14	−0.05	0.26	0.06	−0.04	2	Government Officials Have Manipulated Stock Prices to Hide Scandals
MIST_1	−0.06	0.22	0.05	−0.02	2	A Small Group of People Control the World Economy by Manipulating the Price of Gold and Oil
MIST_32	−0.10	0.31	0.13	0.00	2	The Government Is Conducting a Massive Cover-Up of Their Involvement in 9/11
MIST_20	0.09	0.05	0.44	0.01	3	New Study: Left-Wingers Are More Likely to Lie to Get a Higher Salary
MIST_8	0.08	0.09	0.26	0.00	3	Climate Scientists' Work Is 'Unreliable', a 'Deceptive Method of Communication'
MIST_16	0.01	0.10	0.39	0.05	3	Left-Wingers Are More Likely to Lie to Get a Good Grade
MIST_70	0.14	−0.04	0.00	0.38	4	Majority in US Still Want Abortion Legal, with Limits
MIST_74	0.08	0.00	0.04	0.32	4	Most Americans Say It’s OK for Professional Athletes to Speak out Publicly about Politics
MIST_94	0.06	0.02	0.02	0.30	4	United Nations Gets Mostly Positive Marks from People Around the World

A metric invariance analysis for EGA using permutation tests (Jamison et al., 2022) was conducted using sex, mean age, and mean education as grouping variables. None of the items exhibited a significant (*p* < .05) difference in network loadings across the tested groups, suggesting that the 16 items selected using the EGA framework work similarly irrespective of sex, age, and education (see Supplement S19 for an overview).

**Fig. 8:**
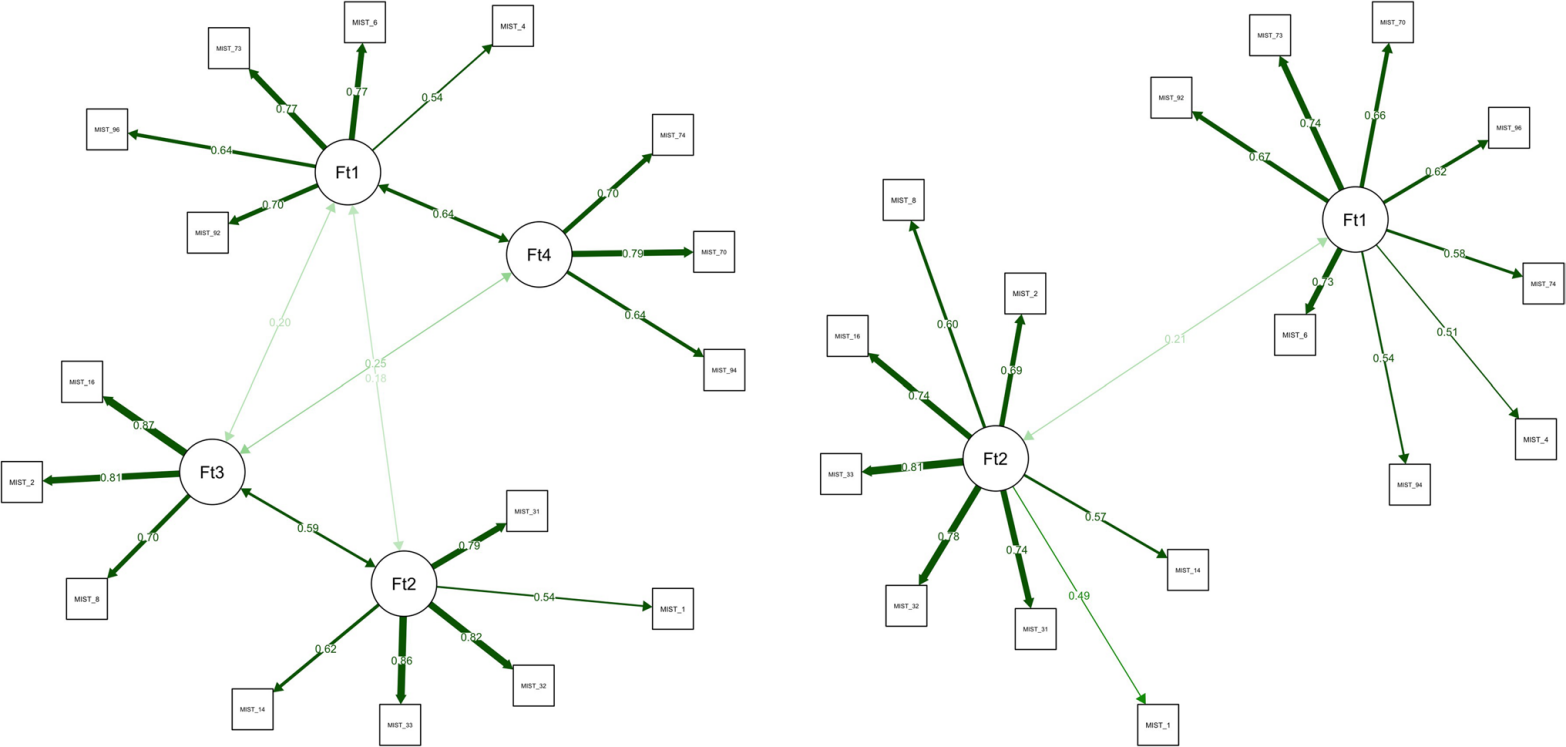
Plot of the confirmatory factor model estimated using the EGA four-factor structure (left) and the theoretical two-factor structure (right)

The fit of the four-dimensional structure estimated via EGA was compared to the fit of the two-factor structure of real and fake news items using the *total entropy fit index* (Golino, Moulder, et al., 2020a), and two traditional factor-analytic fit measures (*CFI* and *RMSEA*). To compute the traditional factor-analytic fit indices, a confirmatory factor analysis was implemented using the *WLSMV* estimator for each structure (see Fig. 8). Table 5 shows that the *EGA four-factor* structure presented the lowest *TEFI* and *RMSEA*, and the highest *CFI*, suggesting that the four-factor first-order dimensions estimated via EGA fit the data better than the theoretical two-factor structure, although the two-factor structure also has an acceptable fit. The Satorra (Satorra, 2000; Table 6) scaled difference test also showed that the *EGA four-factor* structure is preferable to the theoretical two first-order factor structure.

**Table 5:** Comparison of fit indices of the EGA four-factor model and the theoretical two-factor model

Structure	*TEFI*	*CFI*	*RMSEA*
EGA four-factor	−14.27	0.97	0.03
Theoretical two-factor	−11.77	0.91	0.05

**Table 6:** The Satorra scaled difference test comparing the EGA four-factor structure to the theoretical two first-order factor structure

Structure	*Df*	*Chisq*	*Chisq*_Diff_	*Df*_Diff_	*p*
EGA four-factor	98	112.32			
Theoretical two-factor	103	203.49	29.73	5	< .001

Two different traditions were used to select a subset of items, one relying on traditional techniques (EFA and IRT) and another relying on modern network psychometric methods (EGA). Looking at the item stability and structural consistency of the dimensions between the two, we found that the MIST-16 EGA items are stable and consistent, indicating that the four dimensions estimated using exploratory graph analysis are robust and likely to be identified in independent samples. The 20 items selected using EFA/IRT were less robust in terms of stability (see Supplement S19: EGA Metric Invariance Tests). The low stability for some of the items of MIST-20 might indicate that there are a higher or lower number of dimensions underlying the data. The parametric bootstrap EGA analysis (with 500 iterations) of the MIST-20 items indicates that two dimensions are estimated in 21.0% of the bootstrapped samples, three dimensions in 68.2%, and four dimensions in 10.0%. The item stability of the most common structure (three dimensions, see Supplement S20) reveals that the items are relatively stable, but still not as stable as the MIST-16 EGA items. A comparison of the three-dimensional structure estimated using EGA in the MIST-20 items with the theoretical two-factor structure (see Table 7) shows that the three-factor solution performs slightly better, since it presents lower *TEFI* and *RMSEA*, and higher *CFI*.

**Table 7:** Fit of the three- and two-dimensional structures of the MIST-20 items

Structure	*TEFI*	*CFI*	*RMSEA*
MIST-20 EGA three-factor	−20.70	0.963	0.029
MIST-20 Theoretical two-factor	−16.93	0.955	0.032

### Discussion

In Study 1, we generated 413 news items using GPT-2 automated item generation for fake news, and trusted sources for real news. Through two independent expert committees, we reduced the item pool to 100 items (44 fake and 56 real). We then combined item response theory with factor analysis to reduce the item set to the 20 best items for the MIST-20 and the 8 best items for the MIST-8. We found that the final items demonstrate good reliability. In an initial test of validity, we found strong concurrent validity for both the MIST-8 and the MIST-20 as evidenced by their strong associations with the COVID-19 fact-check (a headline evaluation task) and the DEPICT deceptive headline recognition task (a social media post reliability judgment task). Moreover, we found that both the MIST-20 and the MIST-8 outperformed the combined model of the CMQ, BSR, and CRT, when explaining variance in fact-check and DEPICT scores, evidencing incremental validity. This study provides the first indication that both the MIST-20 and MIST-8 are psychometrically sound, and can explain and test misinformation susceptibility above and beyond the existing scales. Finally, we also presented an alternative approach to item selection, namely one based on EGA that uses network psychometrics to identify the best partition of the multidimensional space, combined with a bootstrap analysis of item and dimensional stability (structural consistency), to identify a set of highly stable items with moderate or high network loadings, leading to the selection of 16 items measuring four dimensions of misinformation susceptibility.

## Study 2: Validation—Confirmatory analyses, nomological net, and national norms

Study 2 sought to consolidate and extensively test the psychometric soundness of the newly developed MIST-20, MIST-16, and MIST-8 scales. Across five large samples with nationally representative quotas from two countries (US, UK) and three different recruitment platforms (CloudResearch, Prolific, and Respondi) we pursued three goals. First, we used structural equation modeling and reliability analyses to probe the structural stability, model fit, and internal consistency of the MIST across different empirical settings. Second, we built an extensive nomological network and examined both the correlation patterns and the predictive power of the MIST to demonstrate convergent, discriminant, and incremental validity. Third, we capitalized on the representativeness of our samples to derive national norms for the general population (UK, US) and specific demographic (UK, US) and geographical subgroups (US).

### Method: MIST-20/MIST-8

#### Participants

As part of our EFA/IRT validation study, we collected data from four samples with nationally representative quota (*N*_total_ = 8310, *N*_clean_ = 6461).^21^ Sample 2A was a US sample (*N* = 3692) with interlocking age and gender quota (i.e., each category contains a representative relative proportion of the other category) accessed through *Respondi,* an International Organization for Standardization (ISO)-certified international organization for market and social science research (for previous applications see, e.g., Dür & Schlipphak, 2021; Heinsohn et al., 2019; Roozenbeek, Freeman, et al., 2021a). After excluding incomplete cases and participants outside of the quota, 3479 participants were considered for analysis. Sample 2B was a US sample with nationally representative age, ethnicity, and gender quota (*N* = 856) recruited through *CloudResearch* (formerly *TurkPrime*), an online research platform similar to MTurk but with additional validity checks and more intense participant pool controls (Buhrmester et al., 2018; Litman et al., 2017). After excluding all participants who failed an attention check, were underage, did not reside in the United States, did not complete the entire study, completed the study in ≤ 10 minutes, or were a second-time participant, 510 participants remained.^22^ Sample 2C was a UK sample (*N* = 2517) based on nationally representative interlocking age and gender quota recruited through *Respondi*. After excluding incomplete cases and participants outside of our quota criteria, 1227 participants were retained. Lastly, sample 2D was a UK sample (*N* = 1396) with nationally representative age and gender quota recruited through *Prolific.* Excluding all entries that fell outside of our quota criteria and all incomplete entries resulted in an analysis sample of 1245 participants.

In line with the best practices for scale development to recruit at least 300 participants per sample (Boateng et al., 2018; Clark & Watson, 1995, 2019; Comrey & Lee, 1992; Guadagnoli & Velicer, 1988) and for being highly powered (power = .90, α = .05) to detect the smallest effect size of interest (*r* = .10, needed *N* = 1046; Anvari & Lakens, 2021; Funder & Ozer, 2019; Götz, Gosling, et al., 2022), Samples 2A, 2C, and 2D exceed the size requirements. Sample 2B was highly powered (power = .90, α = .05) to detect effect sizes *r* of .15 (needed *N* = 463). Power analyses were completed using the *pwr* package in R (Champely et al., 2021).

Detailed demographic breakdowns of all samples are shown in Table 1.

#### Procedure and measures

All participants were invited to take part in an online survey through the respective research platforms. After providing informed consent, all participants provided basic demographic information and completed the MIST-20 and—depending on their sample group—a select set of additional psychological measures (for a detailed description of all constructs assessed in each sample group, see Table 1). All participants received financial compensation in accordance with platform-specific remuneration standards and guidelines on ethical payment at the University of Cambridge. Participants in Samples 2A, 2B, and 2C were paid by the sampling platform directly, while participants in Sample 2D received 2.79 GBP for a 25-minute survey (6.70 GBP per hour). All data collections were approved by the Psychology Research Ethics Committee of the University of Cambridge (PRE.2019.108, PRE.2020.034, PRE.2020.086, PRE.2020.120).

#### Analytical strategy

We adopted a three-pronged analytical strategy. First, we computed reliability estimates and conducted confirmatory factor analyses for each subsample, seeking to reproduce, consolidate, and evaluate the higher-order model derived in Study 1. Second, in an effort to establish construct validity (Cronbach & Meehl, 1955; Strauss & Smith, 2009), we pooled the constructs assessed across our four validation samples to build a comprehensive, theory-driven, and preregistered (Sample 2B) nomological network. To this end, we cast a wide net and included (1) concepts that should be meaningfully positively correlated with MIST scores (convergent validity; i.e., *DEPICT Balanced Short Form*; Maertens et al., 2021; *Go Viral! Balanced Item Set*; Basol et al., 2021), expecting a high positive Pearson *r* correlation ([0.50, 0.80]), (2) concepts that should be clearly distinct from the MIST (discriminant validity; i.e., *Bullshit Receptivity Scale*; BSR; Pennycook et al., 2015; *Conspiracy Mentality Questionnaire*; CMQ; Bruder et al., 2013), expecting a low to medium negative correlation with the MIST (Pearson *r* = [−0.50, −0.20]), and (3) an array of prominent psychological constructs of general interest (i.e., personality traits, attitudes, and cognitions including the *Big Five*, *Dark Tetrad*, *Moral Foundations*, *Social Dominance Orientation*, *Ecological Dominance Orientation*, religiosity, self-esteem, political cynicism, numeracy, and trust in various public institutions and social agents) for which no a priori expectations were formulated. Third, we leveraged the size and representativeness of our samples to establish norm tables for the US and UK general populations as well as specific demographic and geographical subgroups.

### Method: MIST-16

#### Participants

We also collected a new dataset (Sample 2E; November 2022) with the best items per dimension that were identified using the EGA approach (the MIST-16). The dataset was collected using Respondi/Bilendi, in a nationally representative quota sample (*N* = 1213) of adults from the US. The sample composition was as follows: 54% identifying as female (44% male, 2% nonbinary), 33% between 18 and 34 years, 31% between 35 and 54 years, and 36% between 55 and 75 years; 24% of the participants reported coming from the Midwest (Illinois, Indiana, Iowa, Kansas, Michigan, Minnesota, Missouri, Nebraska, North Dakota, Ohio, South Dakota, and Wisconsin), 17% from the Northeast (Connecticut, Maine, Massachusetts, New Hampshire, Rhode Island , Vermont, New Jersey, New York, and Pennsylvania), 40% from the South (Florida, Georgia, Maryland, North Carolina, South Carolina, Virginia, West Virginia, Delaware, Alabama, Kentucky, Mississippi, Tennessee, Arkansas, Louisiana, Oklahoma, and Texas), and 20% from the West (Montana, Wyoming, Colorado, New Mexico, Idaho, Utah, Arizona, Nevada, Washington, Oregon, California, Alaska, and Hawaii) of the country.

#### Analytical strategy

Exploratory graph analysis—as well as hierarchical EGA (Jimenez et al., 2022)—was applied to the MIST items. The advantage of using hierarchical EGA (Jimenez et al., 2022) on the US representative quota sample collected (using the best MIST items identified in the first stage of EGA analysis) is that as the sample size increases, there is a realistic chance of EGA estimating a structure reflecting general factors instead of first-order factors, if the dimensions are hierarchical or form a generalized bifactor structure. Therefore, the item stability and structural consistency of the first-order factors were computed using a hierarchical EGA (Jimenez et al., 2022) version of bootstrap exploratory graph analysis (Christensen & Golino, 2021a).

We would like to note that the MIST-16 was developed and validated *after* the samples from the other validation (Studies 2A–2D) and application (Study 3) studies were collected, due to the emergence of new psychometric methods. As the MIST-16 is not a subset of the MIST-20, we do not have the same nomological net and intervention evaluation data available for the MIST-16. However, as the correlation (in Study 1) between the MIST-20 and MIST-16 item sets is large, *r* = .81, 95% CI [.77, .84], *p* < .001, we can expect the MIST-20 results to be a close approximation.

### Results: MIST-20/MIST-8

#### Internal consistency

For each sample, we employed SEM to assess model fit—examining both a basic first-order model with two distinct factors (i.e., real news detection, fake news detection; without allowing the factors to correlate) and a theoretically derived higher-order model (Markon, 2019; Thurstone, 1944; which establishes a relationship between the two factors) in which both first-order factors load onto a general second-order veracity discernment factor. We then calculated reliability estimates using internal consistency measures (inter-item correlations, item-total correlations, and McDonald’s ω). We used the *lavaan* package for SEM in R (Rosseel, 2012).

**Fig. 9:**
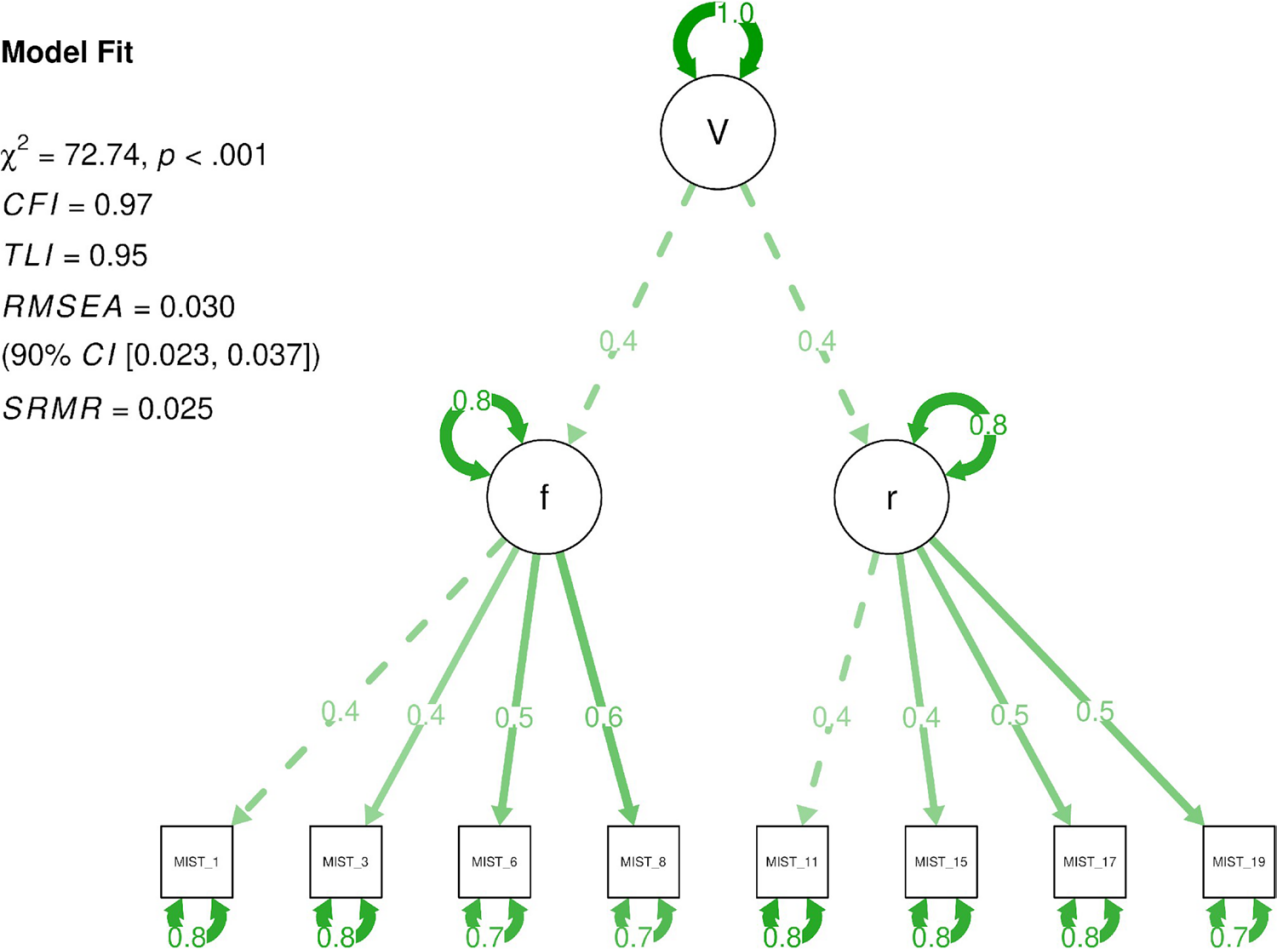
Plot of higher order MIST-8 SEM model in Sample 2A (*N* = 3479)

In keeping with our theoretical conceptualization of the MIST—with a general ability factor of veracity discernment, and two subordinate factors capturing real news and fake news detection, respectively—we fitted a higher-order model (Markon, 2019; Thurstone, 1944) in which both first-order factors load onto a general second-order veracity discernment factor (see Fig. 9). We first did this with Sample 2A (US quota sample from *Respondi*). Consistent with conventional guidelines (*RMSEA*/*SRMR* < .10 = acceptable; < .06 = excellent; *CFI*/*TLI* > .90 = acceptable; > .95 = excellent; Clark & Watson, 2019; Finch & West, 1997; Hu & Bentler, 1999; Pituch & Stevens, 2015; Schumacker et al., 2015), the model fits the data adequately (MIST-20: *CFI* = .90, *TLI* = .89, *RMSEA* = .041, *SRMR* = .040; MIST-8: *CFI* = .97, *TLI* = .95, *RMSEA* = .030, *SRMR* = .025).^23^ We note that the χ^2^ goodness-of-fit test was significant—signaling lack of fit (MIST-20: χ^2^ = 1021.86, *p <* .001; MIST-8: ; χ^2^ = 72.74, *p <* .001). However, this should be interpreted with caution, as the χ^2^ is a test of perfect fit and very sensitive to sample size. As such, as sample sizes approach 500, χ^2^ is usually significant even if the differences between the observed and model-implied covariance matrices are trivial (Bentler & Bonett, 1980; Curran et al., 2003; Rosellini & Brown, 2021). Taken together, the findings thus suggest an adequate model fit for the theoretically derived higher-order model.

Importantly, this model also yielded better fit than a traditional basic first-order model (with two distinct fake news and real news factors; MIST-20: χ^2^ = 1027.17, *p* < .001, *CFI* = 0.90, *TLI* = 0.89, *RMSEA* = 0.041, *SRMR* = 0.041; MIST-8: χ^2^ = 99.46, *p <* .001, *CFI* = 0.95, *TLI* = 0.93, *RMSEA* = 0.035, *SRMR* = 0.035). A likelihood-ratio test of the higher-order model versus the first-order model (which did not include a correlation between the two factors) was significant for both the MIST-20 and the MIST-8 (MIST-20: ∆χ^2^ = 5.35, *p* = .021, MIST-8: ∆χ^2^ = 26.29, *p* < .001), indicating a better fit for the higher-order model.

##### Sample comparison

Across all four samples, we successfully reproduced the original higher-order model, with parameters indicating good fit, as well as good internal consistency in all four samples (see Table 8 for a complete overview).^24^ A similar fit is found between the US *Respondi* and UK *Respondi* samples, indicating that the MIST works similarly in the UK as it does in the US.^25^ Meanwhile, larger differences are found between the US *Respondi* and the US *CloudResearch samples*, and between the UK *Respondi* and the UK *Prolific* samples, indicating that sampling platform plays a larger role than nationality when administering the MIST even when using representative quota sampling.

**Table 8:** Model fit overview

**MIST-20**												
Samp.	Plat.	Pop.	χ²	*p*	*CFI*	*TLI*	*RMSEA*	95% CI		*SRMR*	ω_tot_	3F
								*LL*	*UL*			
2A	R	US	1021.86	< .001	0.90	0.89	0.041	0.039	0.044	0.040	0.76	*
2B	C	US	264.66	< .001	0.92	0.91	0.035	0.027	0.043	0.051	0.75	⚬
2C	R	UK	473.56	< .001	0.91	0.90	0.041	0.037	0.046	0.049	0.81	***
2D	P	UK	432.12	< .001	0.86	0.85	0.038	0.034	0.042	0.045	0.70	***
**MIST-8**												
Samp.	Plat.	Pop.	χ²	*p*	*CFI*	*TLI*	*RMSEA*	95% CI		*SRMR*	ω_tot_	3F
								*LL*	*UL*			
2A	R	US	72.74	< .001	0.97	0.95	0.030	0.023	0.037	0.025	0.57	***
2B	C	US	30.32	.048	0.96	0.94	0.036	0.003	0.058	0.040	0.58	*
2C	R	UK	64.13	< .001	0.94	0.91	0.045	0.033	0.058	0.040	0.62	***
2D	P	UK	46.91	< .001	0.93	0.90	0.037	0.023	0.050	0.035	0.55	***

Total *N* = 6461. Samp = sample. Plat = sampling platform. Pop = sample population. CI = confidence interval; LL = lower limit; UL = upper limit. R = Respondi. C = CloudResearch. P = Prolific. ω_tot_ = McDonald’s Omega. 3F reflects whether the three-factor (higher-order) model provided better fit than the two-factor (two-order) model. ⚬ = descriptively better fit but not significant; * *p* < .05, ** *p* < .01, *** *p* < .001

#### Nomological network^26^

##### Convergent validity

As preregistered, in Sample 2B^27^—which was the sample we primarily relied on in constructing the nomological network, as it offered the widest coverage of psychological constructs among our validation samples—the correlation between the general MIST-20 score and the DEPICT Balanced Short Form measure (Maertens et al., 2021) was found to be positive and medium to large, with a significant Pearson correlation of .54 (95% CI [.48, .60], *p* < .001).^28^ The MIST-20 correlation with the *Go Viral!* inventory (Basol et al., 2021) was lower than the estimated value but was significantly correlated, with a Pearson correlation of .26 (95% CI [.18, .34], *p* < .001). Similarly, regarding incremental validity, the additional explained variance in the DEPICT Balanced Short Form measure above and beyond the CMQ and the BSR is at the upper side of our prediction, with an additional 20% of variance explained, whereas with 3% it is under the predicted value for the *Go Viral!* inventory.^29^ For a more detailed account, see Supplement S13. In addition, in Sample 2A, we measured belief in COVID-19 myths, which was significantly positively correlated and within the preregistered strength of convergent validity measures (*r* = −.51, 95% CI [−.55, −.47], *p* < .001).

##### Discriminant validity

As preregistered for Sample 2B, the MIST-20 was moderately negatively correlated with the BSR (*r* = −.21, [−.29, −.13], *p* < .001) and the CMQ (*r =* −.38 [−.45, −.30], *p* < .001). Overall, the correlational pattern of our nomological network supports the construct validity of the MIST, with the MIST being more strongly correlated with the convergent measures than with the discriminant measures (Campbell & Fiske, 1959; Rosellini & Brown, 2021).

##### CRT (Sample 2A)

In line with other studies finding a role for the CRT in misinformation detection (e.g., Pennycook & Rand, 2019), we found a significant correlation between the MIST score and the cognitive reflection test, or CRT (*r* = .29, 95% CI [.26, .32], *p* < .001).

##### AOT (Sample 2A)

We found an even larger significant correlation between the MIST score and actively open-minded thinking or AOT (*r* = .49, 95% CI [.46, .51], *p* < .001).

##### BFI (Sample 2B)

Contrary to our preregistered exploratory hypotheses, in Sample 2B the MIST-20 score was *not* significantly correlated with openness, *r* = .02, 95% CI [−.06, .11], *p* = .594, and agreeableness was *not* negatively correlated with distrust **d**, *r* = .05, 95% CI [−.04, .14], *p* = .255.^30^ The MIST-20 score was also not significantly correlated with agreeableness (*r* = .05, 95% CI [−.04, .14], *p* = .271) or extraversion (*r* = −.07, 95% CI [−.15, .02], *p* = .141), but did significantly correlate with conscientiousness (*r* = .10, 95% CI [.02, .19], *p* = .020) and neuroticism (*r* = −.14, 95% CI [−.23, −.06], *p* = .001).

##### DT (Sample 2B)

The MIST-20 score was negatively correlated with each of the four Dark Tetrad traits: *Machiavellianism* (*r* = −.09, 95% CI [−.17, −.00], *p* = .047), *narcissism* (*r* = −.26, 95% CI [−.34, −.18], *p* < .001), *psychopathy* (*r* = −.30, 95% CI [−.37, −.22], *p* < .001), and *sadism* (−.22, 95% CI [−.30, −.12], *p* < .001). However, contrary to our preregistered exploratory hypothesis, Machiavellianism was *not* negatively correlated with naïvité **n**, *r* = .16, 95% CI [.07, .24], *p* < .001.

##### Trust measures (Sample 2B)

In line with our preregistered exploratory hypotheses, we found that the MIST score *was* correlated with trust in science, *r* = .33, 95% CI [.25, .41], *p* < .001, scientists, *r* = .36, 95% CI [.28, .43], *p* < .001, and mainstream media, *r* = .18, 95% CI [.09, .26], *p* < .001. In addition, we found that trust in doctors, *r* = .36, 95% CI [.28, .43], *p* < .001, journalists, *r* = .19, 95% CI [.11, .27], *p* < .001, and officials, *r* = .09, 95% CI [.00, .17], *p* = .049, was significantly positively correlated, while trust in the government, *r* = −.11, 95% CI [−.20, −.02], *p* = .012, was significantly negatively correlated with the MIST-20. We found no significant correlation for either of the two trust-in-politicians scales, *r*_a_ = −.06, 95% CI [−.14, .03], *p* = .210, *r*_b_ = .07, 95% CI [−.02, .15], *p* = .131.

##### Additional associations

For a summary and discussion of the exploratory analyses of MFQ, SDO, EDO, numeracy, anti-vaccination attitudes, self-esteem, religiosity, trust, ideology, and demographics, please see Supplement S14.

Detailed summary figures separated by outcome category are available in Supplements S10-S12.

#### National norms

We used the *Respondi* samples for each country (i.e., Sample 2A for the US and Sample 2C for the UK) to generate norm tables for general veracity discernment as well as fake news and real news detection.^31^ As can be gleaned from Table 9, the norms for the two countries were very similar, with minor deviations of single score points, further corroborating evidence for the cross-cultural validity of the MIST. Table 10 exhibits norms for the general US population.

**Table 9:** MIST norm score comparison between US and UK samples

Scale	Sample	*Minimum*	1^st^ *Quartile*	*Median*	*Mean*	3^rd^ *Quartile*	*Maximum*
MIST-8							
	US	0	4	6	6	7	8
	UK	0	4	5	5	7	8
MIST-20							
	US	4	11	14	14	17	20
	UK	4	11	13	13	16	20

**Table 10:** MIST-20 general population norms for the United States (*N* = 3479)

V (Veracity discernment)		f (Fake news detection)		r (Real news detection)	
Percentile	Score	Percentile	Score	Percentile	Score
0%	4	0%	0	0%	0
5%	8	5%	3	5%	2
10%	9	10%	4	10%	3
15%	10	15%	5	15%	4
20%	10	20%	5	20%	4
25%	11	25%	6	25%	5
30%	12	30%	7	30%	5
35%	12	35%	7	35%	6
40%	13	40%	7	40%	6
45%	14	45%	8	45%	7
50%	14	50%	8	50%	7
55%	15	55%	8	55%	7
60%	15	60%	9	60%	7
65%	16	65%	9	65%	8
70%	16	70%	9	70%	8
75%	17	75%	9	75%	8
80%	17	80%	10	80%	9
85%	18	85%	10	85%	9
90%	19	90%	10	90%	10
95%	19	95%	10	95%	10
100%	20	100%	10	100%	10

Full norm tables for the US and the UK, including specific norms based on age (US, UK) and geography (US; i.e., 9 census divisions, 4 census regions), as well as means and standard deviations per item, including a per-item comparison between Democrats (US)/liberals (UK) and Republicans (US)/conservatives (UK), are available in Supplement S15.

#### Results: MIST-16

**Fig. 10:**
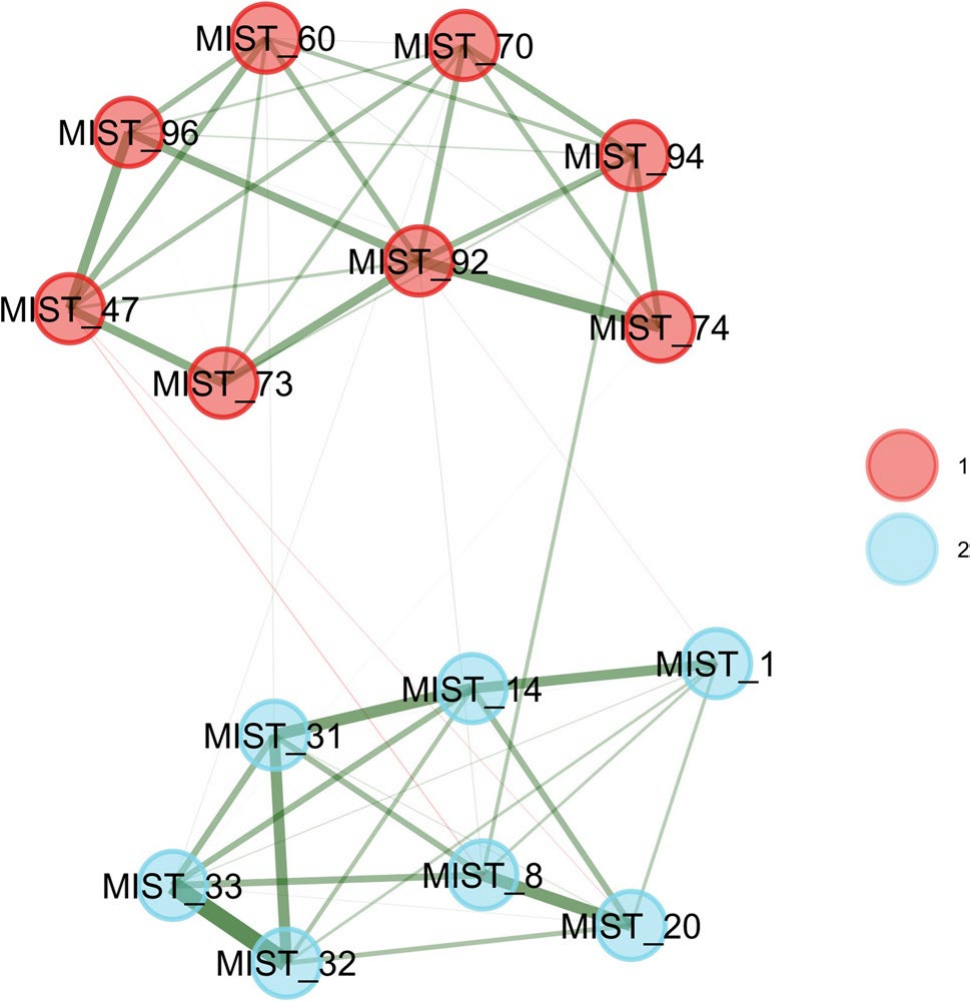
Structure estimated via EGA using the validation sample

**Fig. 11:**
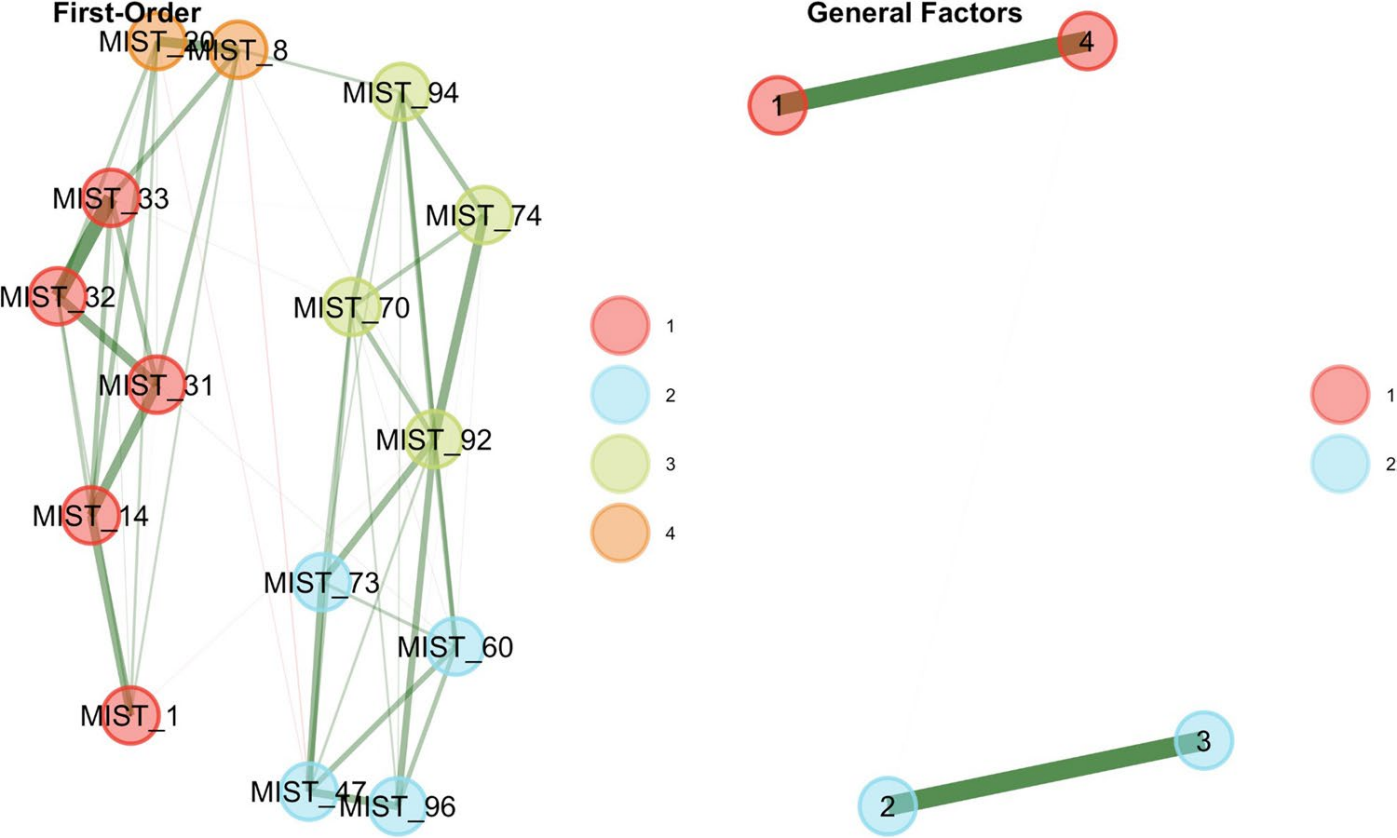
Structure estimated via hierarchical EGA using the validation sample

Exploratory graph analysis was applied to the MIST-16 items, as well as hierarchical EGA (Jimenez et al., 2022).^32^ The item stability and structural consistency of the first-order factors were computed using a hierarchical EGA (Jimenez et al., 2022) version of bootstrap exploratory graph analysis (Christensen & Golino, 2021a).^33^ The traditional EGA technique indeed identified only two dimensions (real and fake news items, see Fig. 10). The hierarchical EGA technique, on the other hand, identified the original four-dimensional (first-order) structure and two general factors (real and fake news items, see Fig. 11).

**Fig. 12:**
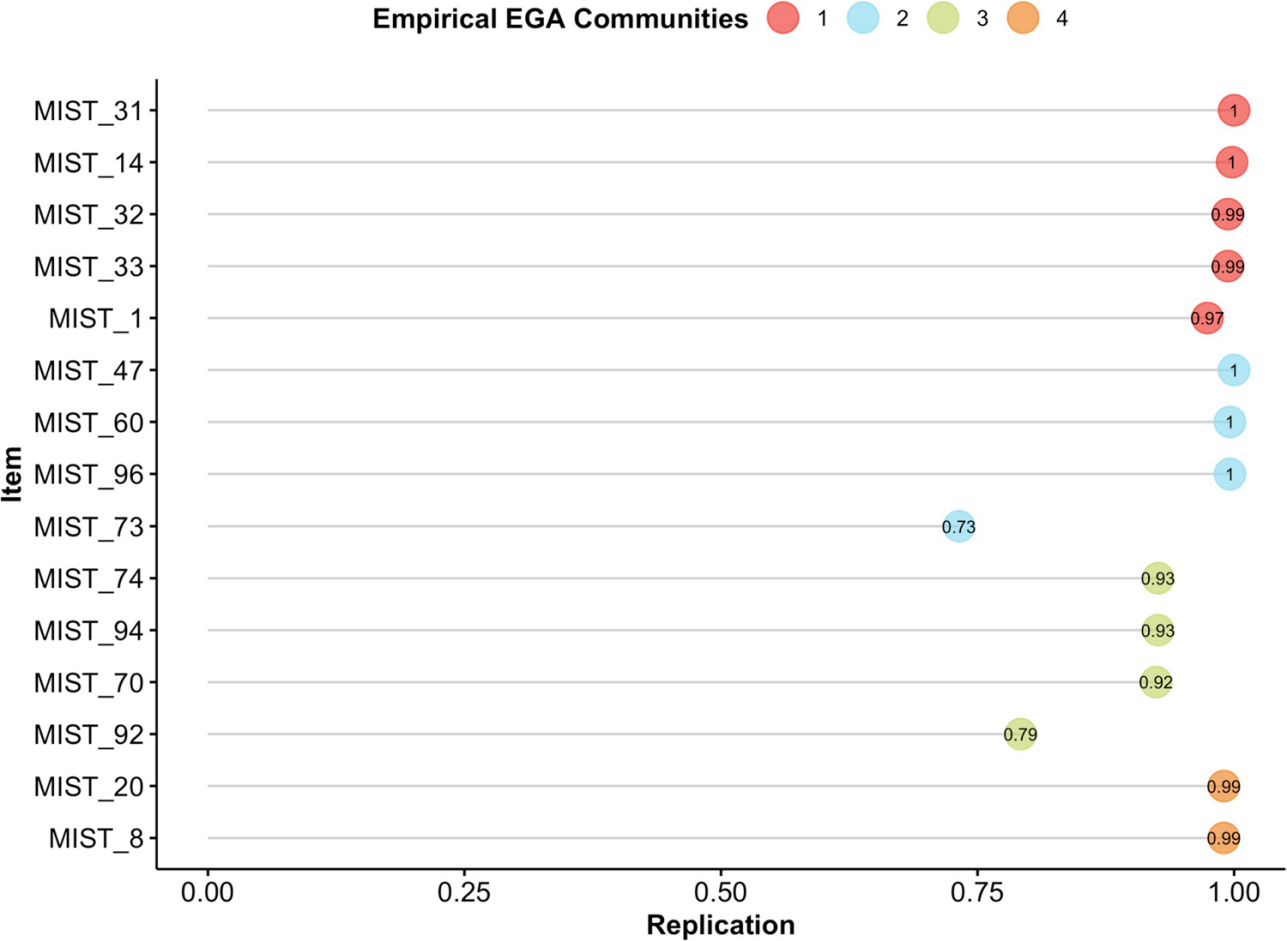
Item stability of the hierarchical EGA first-order structure in the validation sample

A parametric bootstrap EGA using the hierarchical EGA method (Jimenez et al., 2022) showed that the four dimensions are very stable, being estimated in 90.8% of the 500 bootstrapped samples. In terms of item stability, the MIST-16 EGA items presented very high stability, except for item *MIST 73*, which was estimated on their empirical hierarchical EGA first-order dimension in 73% of the bootstrapped samples (see Fig. 12).

### Discussion

In Study 2, we consolidated and expanded the psychometric properties of the MIST. First, we conducted confirmatory factor analyses across four samples with representative quota from the US and the UK, consistently replicating the higher-order structure yielding good model fit and internal consistency for both the MIST-8 and the MIST-20. Next, we constructed an extensive nomological network of the MIST to assess construct validity (Cronbach & Meehl, 1955). As preregistered, and similar to Study 1, in Sample 2B we found a high correlation between the MIST score and the DEPICT misinformation inventory, supporting convergent validity. Similarly, in Sample 2A we found a medium to high negative correlation between the MIST-20 and a COVID-19 misinformation beliefs inventory, further attesting to the measure’s convergent validity. In addition, we demonstrated that both the MIST-8 and the MIST-20 explain considerable extra variance above the existing CMQ and BSR scales (MIST-20: ∆*R*^2^ = 20%, MIST-8: ∆*R*^2^ = 14%), indicating substantial incremental validity (Clark & Watson, 2019). Surprisingly, however, the correlations of each of the MIST, CMQ, and the BSR with the *Go Viral!* items were all low (*r* < .30). Nevertheless, the MIST-20 remained the single best predictor for the *Go Viral!* items, significantly improving the variance explained in a combined model on top of the CMQ and BSR measures (∆*R*^2^ = .03). In terms of discriminant validity, as preregistered, in Sample 2B we observed moderate negative associations between the MIST-20 and the BSR as well as the CMQ. In Sample 2A, we also found preliminary evidence for the role of actively open-minded thinking (AOT) as a potential vehicle for better distinction between fake and real news. This aligns with previous research showing that AOT is related to more critical information source evaluation (Baron, 2019) and decreased susceptibility to fake news (Pennycook & Rand, 2020, Pennycook & Rand, 2021).

Within the realm of trait measures we found relatively small correlations with the core personality traits. Contrary to our expectations, openness, extraversion, and agreeableness were not significantly related to the MIST-20. Meanwhile, conscientiousness exhibited a small positive association. This dovetails well with previous research finding that individuals high in conscientiousness are more likely to read news offline (rather than relying solely on social media; Sindermann et al., 2020) and less likely to share fake news (Lawson & Kakkar, 2021) and engage in conspiracist ideation (Brotherton et al., 2013). We also found a small negative association with neuroticism. As neuroticism is widely understood as a stable predisposition to experience anxiety and fear (Eysenck, 1967; Hofstee et al., 1992; Soto & John, 2017), this is consistent with previous work identifying fear and trait anxiety as positive predictors of conspiracy beliefs (Grzesiak-Feldman, 2013; Swami et al., 2016) as well as other studies finding that those high in neuroticism tend to rely on social media news feeds and are thus more likely to get caught in filter bubbles and echo chambers (Sindermann et al., 2020). Larger correlations were found with the Dark Tetrad personality traits, which were all negatively related to the MIST-20 score. While the links with Machiavellianism, psychopathy, and sadism are novel, the positive association with narcissism dovetails well with previous work demonstrating narcissists’ greater susceptibility to conspiracies (Cichocka et al., 2016; Kumareswaran, 2014).

Meanwhile, in Sample 2E, we successfully validated the psychometric strength of the EGA-based MIST-16, which also showed evidence for two general factors, fake news detection and real news detection, as well as two facets for each. While EGA uses an entirely different approach for item analysis and selection, the convergent outcome of two general factors and the overlap in the item sets between the two methods show that it is possible—using a variety of methodologies—to develop a psychometrically validated misinformation susceptibility test with congruent results. Meanwhile, the EGA data show that EGA is a useful new method psychologists can use to design misinformation detection scales (or indeed, any scale), enlarging the toolkit available for scale development.

All in all, the nomological network largely confirmed the preregistered relationship patterns—thus corroborating the MIST’s construct validity—while at the same time demonstrating new insights that can be gained by using the MIST-20 measure, which may stimulate further research. Finally, we leveraged the large size and national representativeness of our validation samples to produce norm tables for the UK and US general populations as well as distinct demographic subgroups in the UK and the US and geographical subgroups in the US.

## Study 3: Application—A nuanced effectiveness evaluation of a popular media literacy intervention

In Study 3, we demonstrate how the MIST can be used in conjunction with the ***V****e****r****i****f****ication* ***d****o****n****e* framework and norm tables.^34^ We employ the MIST-8 in a simple within-groups pretest /post-test design with the *Bad News Game*, a major media literacy intervention played by over a million people (Roozenbeek & van der Linden, 2019). The *Bad News Game* is based on inoculation theory (van der Linden & Roozenbeek, 2020), and both its theoretical mechanisms and its effects have been replicated multiple times (see, e.g., Maertens et al., 2021; Roozenbeek, Maertens et al., 2021), making it a well-established intervention in the literature as a tool to reduce misinformation susceptibility. We therefore hypothesized that the intervention would improve ***v****eracity discernment* (ability to accurately distinguish real news from fake news), ***r****eal news detection* (ability to correctly flag real news), and ***f****ake news detection* (ability to correctly tag fake news). In addition, we hypothesized that the *Bad News Game* would decreases both **d**istrust (negative judgment bias or being hyper-skeptical) and **n**aïvité (positive judgment bias or believing everything). We used norm tables to establish where the baseline MIST scores of our convenience sample lay.

### Method

#### Participants

We collected data from an online community sample of 4024 participants who played the *Bad News Game* (www.getbadnews.com) between 7 May 2020 and 29 July 2020 and who agreed to participate in the in-game survey. After filtering out participants who did not complete the full study, did not have prior experience with the game, were underage, or entered the study multiple times, and lived outside of the United States, 421 participants remained.^35^ Based on earlier studies evaluating the *Bad News Game* (Maertens et al., 2021; Roozenbeek, Maertens et al., 2021), we aimed to be highly powered (power = .90, α = .05) to detect a Cohen’s *d* effect size of 0.250, which required a sample size of 338, which we exceed in this sample. The power was calculated using the R *pwr* package (Champely et al., 2021).

On average, participants were young (55.58% 18–29 years, 32.30% 30–49, 12.11% over 50), 52.02% identified as female (41.09% male, 6.89% other), and 86% had either a higher education degree or some college experience (see Table 1 for a complete demographics overview). The median ideology on a scale from 1 (*liberal*) to 7 (*conservative*) was 3 (*M* = 2.88, *SD* = 1.39), indicating a slightly left-leaning audience.

#### Procedure and measures

Individuals who played the *Bad News Game* (Roozenbeek & van der Linden, 2019) were invited to participate in the study. The *Bad News Game* (www.getbadnews.com) is a free online browser game in which players learn about six common misinformation techniques over the course of 15 minutes in a simulated social media environment (see Roozenbeek & van der Linden, 2019, for a detailed discussion). In the current study, after providing informed consent, individuals completed the MIST-8 both before and after playing the *Bad News Game*. Participation was completely voluntary, and no rewards, monetary or otherwise, were offered. This study was approved by the Psychology Research Ethics Committee of the University of Cambridge (PRE.2020.120, PRE.2020.136).

#### Analytical strategy

After contextualizing our findings by juxtaposing the sample’s baseline findings to the US general national norms derived in Study 2, we conducted repeated-measures *t*-tests for veracity discernment (*M* = 6.23, *SD* = 1.53) and for the four subcomponents of the MIST—**f**ake news detection (*M* = 3.19, *SD* = 0.92), **r**eal news detection (*M* = 3.04, *SD* = 0.95), **d**istrust (*M* = 0.31, *SD* = 0.63), and **n**aïvité (*M* = 0.46, *SD* = 0.69).

### Results

#### Baseline

We found that our US convenience sample scored higher on the MIST than the US population average for **v**eracity discernment (see Study 2; 1^st^ *Quartile*_Population_ = 4, 1^st^ *Quartile*_Sample_ = 6).^36^

#### Hypothesis tests

##### V—Veracity discernment

**Fig. 13:**
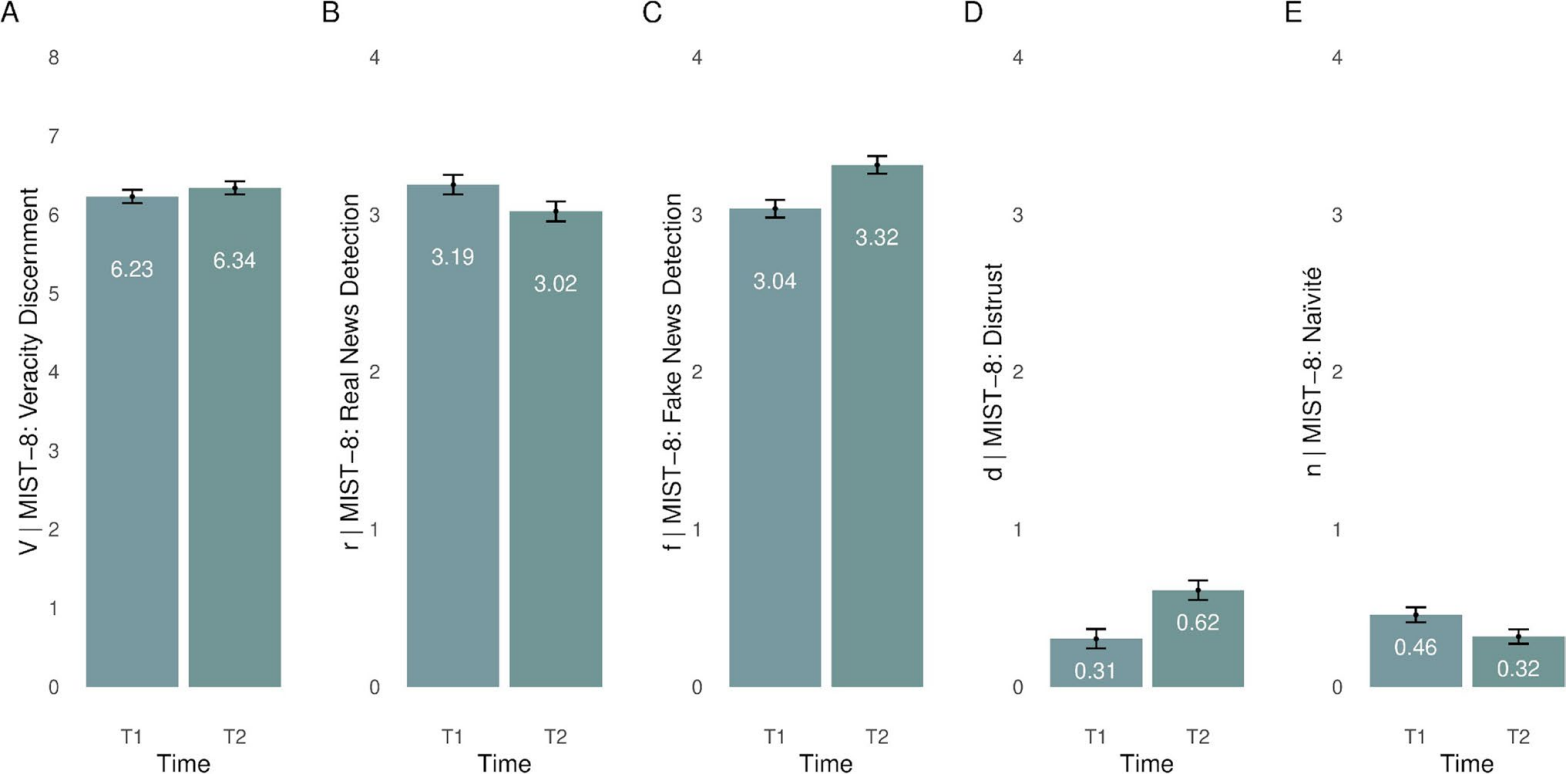
Plot of *Verification* ***d****o****n****e* variables applied to the *Bad News Game* (*N* = 421). T1 = pretest. T2 = post-test

Contrary to our expectations, we did not find a significant effect of veracity discernment post-intervention relative to pre-intervention (*M*_diff_ = 0.11, 95% CI [−0.01, 0.23], *t*(420) = 1.80, *p =* .072, *d* = 0.088, 95% CI [−0.103, 0.279]). See Fig. 13, Panel A for a bar plot.

##### r—Real news detection

While we found an effect of the intervention on real news detection, the effect was in the opposite direction of our prediction (*M*_diff_ = −0.17, 95% CI [−0.26, −0.08], *t*(420) = −3.72, *p <* .001, *d* = −0.181, 95% CI [−0.373, 0.011]). See Fig. 13, Panel B, for a bar plot.

##### f—Fake news detection

In line with our expectations, we did find a positive effect of the intervention on fake news detection (*M*_diff_ = 0.28, 95% CI [0.20, 0.36], *t*(420) = 6.81, *p <* .001, *d* = 0.332, 95% CI [0.138, 0.525]). See Fig. 13, Panel C for a bar plot.

##### d—Distrust

Contrary to our hypothesis, we observed an increase in distrust (*M*_diff_ = 0.31, 95% CI [0.22, 0.40], *t*(420) = 6.94, *p <* .001, *d* = 0.338, 95% CI [0.144, 0.532]). See Fig. 13, Panel D for a bar plot.

##### n—Naïvité

As hypothesized, we did find a significant reduction in naïvité after intervention (*M*_diff_ = −0.14, 95% CI [−0.20, −0.07], *t*(420) = −4.12, *p <* .001, *d* = −0.201, 95% CI [−0.392, −0.008]). See Fig. 13, Panel E for a bar plot.

See Supplement S16 for a detailed summary table with variable descriptive statistics and difference scores.

### Discussion

Traditionally, evaluators of *Bad News Game* (e.g., Roozenbeek & van der Linden, 2019) only looked at a small amount of (ad hoc-created) real news items and focused on participants’ reliability ratings of a large set of fake news items. Study 3 showed that using the MIST in conjunction with the ***V****e****r****i****f****ication* ***d****o****n****e* framework provided novel insights contrary to our expectations. Although trending towards an effect in the expected direction, participants did not become significantly better at general news veracity discernment after playing the *Bad News Game* (*p* = .072). Looking at the MIST facet scales, we did find significant differences in both fake news detection and real news detection. More specifically, we observed that while people improved in the detection of fake news, they also became worse at the detection of real news. Looking further at response biases, we can also see that the *Bad News Game* might increase general distrust in news headlines while also diminishing naïvité. At first sight, these results seem to indicate that the intervention does decrease people’s susceptibility to fake news and reduces general naïvité, but at a potential cost of increased general distrust (hyper-skepticism). Whether this means the intervention works depends on the aim: to decrease susceptibility to misinformation, or to increase the ability to accurately discern real news from fake news. The ***V****e****r****i****f****ication* ***d****o****n****e* framework allows interventionists to start differentiating these important questions both theoretically and empirically, and we encourage researchers and practitioners to use the framework independently of the misinformation susceptibility measure used.

One reason why the pattern for the subordinate factors may be found is that the *Bad News Game* focuses mainly on detecting misinformation and warning people about the threats of misinformation, and is less focused on recognizing real news (Roozenbeek & van der Linden, 2019). In addition, as the evidence shows there may be counteracting effects (increased **d**istrust but also improved **f**ake news detection), the lack of significant effects for the general factor (the discernment variable) may therefore also be due to these counteracting effects, resulting in an effect that is too small to measure with our sample (*N* = 421), especially in the context of a short 15-minute intervention in combination with an 8-item scale. Finally, it is also possible that the intervention may simply not be sufficient to make a large enough impact on a general susceptibility factor.

In addition, as recommended by our framework, these results need to be interpreted in conjunction with the norm tables. The general sample that was recruited was already highly media-literate. The first quartile of the pretest MIST scores was higher than the population average (veracity discernment: 1^st^ Quartile_Population_ = 50% accuracy, 1^st^ Quartile_Sample_ = 75% accuracy). Effects of the intervention might therefore be different with a more representative sample, or for people performing worse during the pretest phase.

The results of this study come with two caveats. First, the MIST-8 was used instead of the MIST-20. As is common for short scales (Rammstedt et al., 2021; Thalmayer et al., 2011)—while maintaining high psychometric quality—the parsimonious MIST-8 is less precise and less reliable than the MIST-20. Since the MIST-20 only takes about 2 minutes to complete, we recommend researchers use the MIST-20 whenever possible. Second, while we were sufficiently powered to detect effect sizes similar to the original evaluation of the intervention (Roozenbeek & van der Linden, 2019), with a sample of 421 participants—as is also reflected in the rather large confidence intervals—we did not have sufficient statistical power to detect smaller nuances (Anvari & Lakens, 2021; Funder & Ozer, 2019; Götz, Gosling, et al., 2022).

The results of this study indicate the importance of looking at misinformation susceptibility in a more holistic way. Applying the ***V****e****r****i****f****ication* ***d****o****n****e* framework, we discovered key new theoretical dimensions that previous research had overlooked. Evaluators of this intervention, and other interventions, can now disentangle and accurately measure the five dimensions of misinformation susceptibility, thereby expanding our understanding of both the underlying mechanisms and the intervention’s practical impact.

## General discussion

We explained the necessity of having a multifaceted measurement of misinformation susceptibility, and based on theoretical insights from previous research, developed the ***V****e****r****i****f****ication* ***d****o****n****e* framework. Then, in three studies and six samples from two countries, we developed, validated, and applied the Misinformation Susceptibility Test (MIST): a holistic test which allows the assessment of *veracity discernment ability*, its facets *fake news detection ability* and *real news detection ability*, and judgment biases *distrust* and *naïvité*.

In Study 1, we derived a development protocol, generated a set of fake news headlines using the GPT-2 neural network—an advanced language-based machine learning algorithm—and extracted a list of real news headlines from neutral and well-trusted sources. Through psychometric analysis using factor analysis and item response theory, we developed the MIST-8, MIST-16, and the MIST-20 tests.

In Study 2, we recruited five samples with nationally representative quota, two each for the US and the UK, from three different recruitment platforms, and followed a multifaceted validation strategy with the aim of gaining insights into the measure’s validity and replicability. First, confirmatory factor analyses consistently favored the higher-order structure and yielded satisfactory properties that suggest high validity and good reliability of both the MIST-8 and the MIST-20. Second, adopting a wide-net approach, we constructed an extensive nomological network. We found the MIST-8 and MIST-20 to be consistently highly correlated with various fact-check tests—the “COVID-19 fact-check” headline evaluation task (Pennycook, McPhetres, et al., 2020) and the and “DEPICT” social media post reliability judgment task (Maertens et al., 2021)—thus signaling convergent validity—while being clearly distinct from the existing Conspiracy Mentality Questionnaire (CMQ) and the Bullshit Receptivity Scale (BSR), hence providing evidence for discriminant validity. The correlation with ad hoc headline evaluation tasks is strong enough to show that they are measures of a similar construct, but it is also weak enough to demonstrate that they are sufficiently distinct. The MIST offers a reliable, standardized, and validated alternative to these ad hoc tests, with high predictive validity for a wide set of scales, as well as norm tables. However, due to the high stability of the MIST, it is possible that the MIST may turn out to be particularly useful for subgroup analyses, and may be less sensitive for the measurement of (small) intervention effects. In addition, the MIST aims to measure *generalized susceptibility* to misinformation, which is not tailored to the skills trained in specific interventions. Therefore, the MIST is not meant to replace ad hoc measures, but can exist in conjunction with them, depending on the outcome variable of interest. Moreover, we presented MIST-20 and MIST-8 norm tables for both the UK and the US based on our large samples with nationally representative quota, which can be used to contextualize effects.

Using a new, modern, psychometric method, namely exploratory graph analysis (EGA; Golino & Epskamp, 2017), we showed a proof of concept of how EGA can be used to help with establishing the factor structure, the item selection, and the validation of scales such as the MIST. In both Study 1 and Study 2 we show how EGA can lead to potentially more stable item selection than when using the traditional EFA and IRT methods, and present an alternative version of the MIST: the MIST-16. Meanwhile, further analyses reveal that EGA can help to detect extra dimensions as facets of the general factors. Interestingly, the validation sample (Sample 2E) showed that a structure with two generalized factors and four facets had the best fit, potentially informing misinformation theorists on further dimensions to explore when researching the nature of misinformation. Meanwhile, it also corroborated more evidence that misinformation susceptibility can be viewed through the lens of two general factors (real news detection, fake news detection), and robustly measured as such. This congruence between these two very different psychometric methods shows the robustness of our psychometric toolkit and the ability for it to produce reliable scales to measure psychological constructs.

In the third and last study, we demonstrated how ***V****e****r****i****f****ication* ***d****o****n****e* and the MIST can be employed in naturalistic settings, in this case to evaluate the general effects of a highly popular inoculation intervention. Employing a validated measure to evaluate interventions in combination with the norm tables—which have not been used in this field before—we were able to uncover new mechanisms behind a well-known media literacy intervention, the *Bad News Game* (Maertens et al., 2021; Roozenbeek & van der Linden, 2019), and highlighted both weaknesses and strengths of this intervention that had not been detected before using the classical methods. For example, while the intervention is typically evaluated by looking at fake news reliability ratings (e.g., Roozenbeek & van der Linden, 2019) without an evaluation framework or norm tables, we were now able to unveil important dynamics between fake news, distrust, and real news detection. Moreover, our approach allowed us to establish that the average participant who chose to participate in the intervention already scored above the norm when completing the pretest. Moreover, for the first time, we were able to disentangle the five dimensions of misinformation susceptibility using a validated and standardized item set, finding unexpected changes in judgment biases as well as in real news detection (which other research does not necessarily find; see Roozenbeek & van der Linden, 2019), which can inspire further research and theoretical development. Nevertheless, we must emphasize that the MIST is a *generalized* measure of susceptibility, relevant for measuring an overarching skill, which is not the sole focus of the *Bad News Game* intervention. For example, there is a wide range of evidence that shows that the *Bad News Game* is effective at improving the detection of specific manipulation techniques that typically underlie misinformation that the participant was trained on (e.g., appeal to emotion, polarizing language; Roozenbeek & van der Linden, 2019; Lewandowsky & van der Linden, 2021). Improvements in those specific skills can be best identified with a tailored measurement instrument rather than a “general” measure such as the MIST.

Overall, these studies show that it is feasible to develop a psychometrically validated measurement instrument for misinformation susceptibility. Moreover, the evidence discussed in the studies, and in particular the analyses of Table 3, Supplement S13, and Supplement S18, show clear evidence for the utility—or indeed superiority—of the new measure compared to other measures in terms of predicting outcomes.

### Implementation

An overview of the MIST-20, MIST-16, and MIST-8 item sets can be found in Supplement S21. For an implementation and scoring guide, please see Supplement S17. The supplements can be found on the OSF repository at https://osf.io/r7phc/.

### Open-Source web application

To facilitate the implementation of the MIST, we programmed an open-source, user-friendly, online version of the MIST-20, called *YourMIST*: an interactive self-assessment tool designed for easy accessibility and repurposing by individuals, researchers, and practitioners. Our implementation of the MIST-20 utilizes the Python programming language and the Streamlit web development module to enable a web-based quiz that provides personalized feedback to users. The tool reports scores for each of the components of the ***V****e****r****i****f****ication* ***d****o****n****e* framework, accompanied by detailed explanations and a comparison with the US and UK population scores. Our web app and the source code are publicly accessible for individual use and adaptation on the OSF repository at https://osf.io/r7phc/.

### Limitations and future research

While we firmly believe that the MIST and ***V****e****r****i****f****ication* ***d****o****n****e* mark a substantial methodological advance in the field of misinformation research (Bago et al., 2020; Batailler et al., 2022; Roozenbeek, Maertens et al., 2021; Rosellini & Brown, 2021; Zickar, 2020), it is of course not without limitations. An inevitable challenge of doing any type of systematic and methodologically rigorous news headline research lies in the fact that what might be real news at one point in time might be outdated at a later point in time, while—albeit admittedly much less likely—what is fake news at one point in time might become true or more credible at a later point in time. Therefore, similar to an IQ test, it may be necessary to update the MIST over time. Nevertheless, in recent studies, the MIST still shows similar validity as it did 2 years ago. To illustrate, in a recent research project by Said et al. (2023, in prep), a new US quota sample was collected through Respondi with 547 respondents, and both the MIST-8 and MIST-20 showed good internal and predictive validity similar to the original sample (see Supplement S7). For example, the fit indices of the MIST sample collected in August 2022 (MIST-20: *CFI* = 0.92, *TLI* = 0.91, *RMSEA* = 0.039, *SRMR* = 0.052) showed similar—and for some indices *better*—fit relative to the sample collected in September 2020 (MIST-20: *CFI* = 0.90, *TLI* = 0.89, *RMSEA* = 0.041, *SRMR* = 0.040). Similarly, the MIST-20 was an even better predictor of performance on the DEPICT deceptive headlines recognition task (Maertens, Roozenbeek, et al., 2021) in the August 2022 (*r* = .64, *p* < .001) sample than it was in the April 2020 sample (*r* = .50, *p* < .001).

Another related limitation concerns the inherent difficulty in the MIST’s cross-cultural application. While we are greatly encouraged by our finding that the MIST appears to be an equally effective measure in the UK as in the US-American cultural context in which it was originally developed, cross-cultural translation poses a challenge. For obvious reasons, a simple and direct translation may not be sufficient. At the same time, while trustworthy news sources from which real news items could be extracted can doubtlessly be identified in any language, at the time the MIST-20 was developed, the GPT-2 (Radford et al., 2019)—the advanced language-based neural network algorithm that we employed to generate fake news items—was mainly trained on English language corpora. Meanwhile, however, an increasing amount of new research and applications has managed to make the GPT-2 work in the context of other languages (see, e.g., de Vries & Nissim, 2020; Guillou, 2020; for promising initial applications in Dutch, Italian, and Portuguese). Moreover, the recent arrival of GPT-3 and GPT-4, which have support for an increasingly wide range of languages, now enables the field to develop non-English adaptations of the MIST that will empower researchers around the globe to capture the complex and multifaceted reality of misinformation spread—and resistance. Even without the GPT-2, researchers can create a database of their own misinformation items and use the same psychometric techniques as outlined in this paper to come to a valid misinformation susceptibility test in any culture. Therefore, we see this paper as a proof of concept on the feasibility of using psychometrics to develop a comprehensive misinformation susceptibility test in any culture.

One other concern that may be raised is that the MIST may be confounded with general news consumption, meaning that those who are more aware of the news may be more likely to score high on the MIST and controlling for this may reduce the MIST’s predictive validity, and that misinformation news engagement is often driven by partisan polarization and outgroup derogation (Osmundsen et al., 2021). To investigate these concerns, we looked at data from a separate study that is currently being prepared, which contains the MIST, the CMQ, and a social media misinformation and manipulative posts discernment test (Maertens et al., 2022, in prep). Looking at these data (*N* = 2220, US quota sample, Respondi), we found that the MIST was the single best predictor for manipulative headline discernment above the CMQ and news consumption (not controlling for news consumption: β = 0.366, *p* < .001, controlling for news consumption: β = 0.362, *p* < .001), that general news consumption was only weakly correlated with MIST performance (*r* = 0.218, *p* < .001), and that news consumption did not have an impact on the MIST’s predictive validity (see Supplement S18). In other words, the MIST discernment score does reflect ecologically valid discernment, and is not confounded by news consumption.

Finally—although based on the consistent results across samples and time points it is unlikely that this has confounded the results—it should be noted that in all studies and with all samples, we have excluded participants who did not complete the entire study up to the analysis of interest. This means that in Study 1, the test–retest reliability may be influenced by the type of participants who participated in the follow-up (i.e., long-term Prolific users), in Study 2 it is possible that the construct validity findings were influenced by excluding participants who dropped out during the study, and in Study 3 it is possible that the evaluation was influenced by some participants dropping out between the pretest and post-test.

We can see many more avenues for future studies using ***V****e****r****i****f****ication* ***d****o****n****e* and the MIST. One example is the implementation of the MIST in geo-psychological studies (Ebert et al., 2021; Rentfrow et al., 2013, 2015) to identify misinformation hotspots and covariates with national, regional, and local levels of misinformation susceptibility. Another strand of research may further deepen our conceptual understanding of media literacy. For example, in light of the current findings, it appears that veracity discernment may encompass both a comparatively stable, trait-like component, and a more malleable skill component. Future studies may more clearly identify this distinction and find ways to best use these insights to devise effective interventions that foster better detection of both fake news and real news, and in turn ultimately lead to greater genuine veracity discernment.

Finally, we identify six immediate use cases for the MIST: (1) to prescreen participants for studies, (2) as a covariate to investigate subgroups (e.g., that are highly susceptible to misinformation), (3) as a control variable in a model, (4) to map geographical regions to identify misinformation susceptibility hotspots, (5) to identify brain regions linked to misinformation susceptibility, and (6) to evaluate interventions. In addition, we would like to encourage the use of the ***V****e****r****i****f****ication* ***d****o****n****e* framework as a general method to look at misinformation susceptibility and intervention effects more holistically, independent of the measure used: indeed, we would encourage practitioners to use the framework with any tests.

### Conclusion

Researchers lack a unifying conceptualization of misinformation susceptibility and too often use unvalidated measures of misinformation susceptibility. We therefore developed a new overarching, unifying and multifaceted interpretation framework (i.e., ***V****e****r****i****f****ication* ***d****o****n****e)* and a new, thoroughly validated measurement instrument based on this framework (i.e., the Misinformation Susceptibility Test; MIST). The current paper acts as a blueprint of integrated theory and assessment development, and opens the door to standardized and comparative misinformation susceptibility research. Both researchers and practitioners can now make a thorough evaluation of media literacy interventions by comparing MIST scores using the norm tables and the ***V****e****r****i****f****ication* ***d****o****n****e* framework. The use of our standardized and psychometrically validated instrument allows for a comprehensive evaluation, and also permits holistic comparison studies and tables to be compiled reporting all five ***V****e****r****i****f****ication* ***d****o****n****e* scores. Practitioners in turn can use these scores and comparisons to choose interventions that best fit their needs. ***V****e****r****i****f****ication* ***d****o****n****e* and the MIST can be employed across a range of psychological disciplines, ranging from cognitive neuroscience to social and personality psychology, to reveal the psychological mechanisms behind susceptibility to misinformation or to test the outcome of interventions.

### Supplementary Information

ESM 1 (DOCX 20 kb)

## Footnotes

1 It should be noted that recent research also provides evidence for an alternative perspective, namely that the spread of misinformation could be driven more by an emotional dimension than a veracity dimension (Cinelli et al., 2020).

2 While there are models that take into account the baseline plausibility of each item, they still do not reveal what construct each item is measuring. In other words, there may still be unexplained variability even when controlling for baseline plausibility, such as issues with item stability, and different effect sizes between item sets in intervention studies.

3 We chose the binary coding approach (i.e., true versus false headline) because it allows us to create a straightforward and easy-to-interpret structure with either a correct or an incorrect response for each item, which is also easy to implement and analyze in a performance-based IRT model, without compromising on quality (e.g., in Studies 1–2 we validated the MIST with items that are administered with Likert scales, providing evidence for its broader predictive validity).

4 Note that **d**istrust and **n**aïvité were not included in the psychometric scale development protocol, but only added later on as a post hoc calculation. The factor structure used for the scale development using EFA/IRT analyses can be found in Fig. 9, and the structure used for the EGA-based scale can be found in Fig. 7.

5 For a step-by-step guide on how to set up the GPT-2 to use as a psychometric item generator, see the tutorial paper by Götz et al. (2023), as well as the useful blog posts by Woolf (2019), Nasser (2020), and Curley (2020).

6 MBFC is an independent fact-checking platform that rates media sources on factual reliability as well as ideological bias. At the time of writing, the MBFC database lists over 3700 media outlets and its classifications are frequently used in scientific research (e.g., Bovet & Makse, 2019; Chołoniewski et al., 2020; Cinelli et al., 2021).

7 Three out of six no longer receive the maximum score, and are now considered to have a *center-left* bias, and score between *mostly factual* and *highly factual* reporting: World Press Review (*mostly factual*, *center-left*), MapLight (*highly factual*, *center-left*), and JStor Daily (*highly factual*, *center-left*). This reflects both the dynamic nature of news media and the limits of the classification methodology used.

8 We used three-fourths as a criterion instead of 100% consensus because, as experts, we may be biased ourselves, and therefore we also accepted items where only one expert did not agree. If less than 120 items would remain, then the Phase 1 item generation process would be restarted.

9 We preregistered that we would split the sample in half for exploratory analyses and confirmatory analyses. However, we used the full Study 1 sample for exploratory analyses instead and conducted a new study with a fresh sample (Study 2) for the confirmatory analyses. This more rigorous and more conservative approach was chosen to boost power and increase the quality of the initial item selection.

10 We looked at the difference in demographics between T1 and T2 Prolific users. While we found no noteworthy differences in age (*M*_T1_ = 33.20, *M*_T2_ = 35.76) or educational attainment rates, (T1: 38.88% with bachelor’s degree, T2: 41.52% with bachelor’s degree), the percentage of female participants rose somewhat during the follow-up (T1: 55.50% male, T2: 39.72% male).

11 All headlines can be found in Supplement S1.

12 We chose to have a follow-up to be able to measure changes in the MIST score over the medium long term. We found a period of eight months fitting for this purpose.

13 The factorability of the data was tested via the Kaiser–Meyer–Olkin (KMO) measure of sampling adequacy and Bartlett’s test of sphericity using R and the *EFAtools* package (Steiner & Grieder, 2020). Both tests indicated excellent data suitability (Bartlett’s χ^2^ = 12,896.84, *df* = 4950, *p <* .001; *KMO* = .831) according to established guidelines (Carpenter, 2018; Tabachnick & Fidell, 2007).

14 These five factors are in line with the criteria set out in the preregistration, as they have both (i) an eigenvalue > 1 and (ii) an eigenvalue larger than the simulated value (above the line of randomly generated data).

15 When using EFA with a promax rotation, there is some evidence for two factors for the fake news items and two factors for the real news items, bringing up a total of four factors, but its pattern and meaning is unclear. This alternative structure will be further explored in the EGA section.

16 While we chose to adhere to the more traditional methods for estimating and rotating factors in EFA, we acknowledge that recent research provides arguments for the use of ML estimation and oblique rotations (Goretzko et al., 2021), and specifically ULS estimation (using the tetrachoric correlation matrix) for dichotomous variables (see Shi et al., 2018). We provide an alternative, modern approach to item selection based on EGA in the section below.

17 We note that some researchers argue that the focus on reliability can reduce the content validity of the scale, as there may be relevant items with weaker loadings (e.g., Flake et al., 2017). However, as no items were removed, this is not a concern for this study.

18 As can be glimpsed from the final set, the misinformation items contain certain words and topics that are more often linked to manipulative content, such as “control/manipulate/cause,” “vaccine/virus,” and “government.” These topics were already present in the sample items given to the GPT-2—which led to more of these topics being present in the original fake news item pool than in the real news item pool. This thus represents a feature that was present since the first phase of the development and is not just a consequence of a later selection by the experts or elimination based on factor loadings.

19 It must be noted that at T2, participants only completed the 20-item MIST, while at T1 participants had to categorize 100 items, with slightly different question and response framings (see full Qualtrics layouts and question framings in the OSF repository: https://osf.io/r7phc/). We expect the actual test–retest correlation to be higher.

20 Full model output for the MIST-8 and MIST-20 linear models can be found in Supplement S8. Full analysis scripts can be found in the OSF repository: https://osf.io/r7phc/.

21 Surveys 2A, 2C, and 2D were designed as part of a separate research project which featured the MIST-20 as an add-on. Survey 2B was designed specifically for this project.

22 This is a slight deviation from the preregistration, as we added incomplete entries, second entries, participants that completed the survey in ≤ 10 minutes, and participants who failed *any* attention check (instead of *both*) to the exclusion criteria, thus adopting a more rigorous and conservative exclusion approach than we had preregistered. These additional exclusions were to ensure high-quality data.

23 We acknowledge that there is a discussion in the literature on defining new (dynamic) fit values depending on the specific model tested (see McNeish & Wolf, 2021). For example, simulations using the *ezCutoffs* (Schmalbach et al., 2019) package indicate we would need a *CFI* and a *TLI* of larger than 0.99 for excellent fit, in conjunction with an *RMSEA* of smaller than 0.04/0.03 (MIST-8/MIST-20) and an *SRMR* smaller than 0.03. However, as the new cutoff values are still under consideration and not well established, we focused on the conventional and—in this case also—preregistered cutoff values for our evaluation.

24 Supplement S9 includes model plots for both the MIST-20 and MIST-8 for all samples.

25 We would like to stress that this does not imply measurement invariance and would like to caution researchers to compare results directly between countries. The current data indicate that the MIST works in the US and the UK and likely measures the same latent construct, but it does not mean that the results are *directly* comparable. We recommend researchers and practitioners keep the focus on comparisons within instead of between countries. For a detailed discussion about cross-cultural generalizability please see Deffner et al. (2022).

26 This section focuses on the nomological network of the general ability factor (veracity discernment) of the MIST-20. However, we have also constructed nomological networks for the subcomponents of the MIST as well as the MIST-8. For parsimony’s sake, these are reported in Supplements S10-S12.

27 Some variables were only analyzed in specific samples, as not all variables were present in all datasets.

28 See https://aspredicted.org/nx7xu.pdf for the preregistration (Sample 2B).

29 It must be noted that the Go Viral inventory is not a validated measurement instrument. Results should be interpreted in light of this.

30 The lack of a significant correlation between the MIST score and openness is somewhat surprising given the strong correlation between the MIST and the AOT score, indicating that openness as measured in the Big Five is not the same as open-minded thinking as measured by the AOT.

31 We chose to create the norm tables based on the Respondi samples instead of pooling all samples, as through recent projects we found some evidence indicating that Respondi samples provide more representative levels of *numeracy*, *education*, and *ideology* than Prolific, and our experience with CloudResearch is limited.

32 Due to an error in the Qualtrics system, only 15 items were presented to the participants. Item MIST 16 (*Left-Wingers Are More Likely to Lie to Get a Good Grade*) was left out of the data collection system.

33 As pointed out earlier, the advantage of using hierarchical EGA (Jimenez et al., 2022) on the US representative quota sample (collected using the MIST-16 EGA items) is that as the sample size increases, there is a meaningful chance that the EGA estimates a structure reflecting general factors instead of first-order factors, if the dimensions are hierarchical or form a generalized bifactor structure.

34 A MIST implementation guide explaining how researchers and practitioners can set up the MIST in their studies as well as how to calculate the *Verification done* (Vrf dn) scores can be found in Supplement S17. An example Qualtrics survey and a score calculation R script are available in the OSF repository: https://osf.io/r7phc/.

35 We restricted our sample to US residents, as we did not have a UK filter and have not yet validated the MIST in any other country.

36 We found similar results when looking at fake news detection (1^st^ *Quartile*_Population_ = 2, 1^st^ *Quartile*_Sample_ = 3) and real news detection (1^st^ *Quartile*_Population_ = 2, 1^st^ *Quartile*_Sample_ = 3).
