# Supplementary material for: The Misinformation Susceptibility Test (MIST): A psychometrically validated measure of news veracity discernment
Source: Behav Res Methods. 2023 Jun 29;56(3):1863–99. doi: 10.3758/s13428-023-02124-2 (PMC10991074; doi:10.3758/s13428-023-02124-2)
Supplement: Supplementary file 1 — (DOCX 20 kb) [file 13428_2023_2124_MOESM1_ESM.docx]

The supplement, data, and analysis scripts that support this paper’s findings are openly available on the Open Science Framework (OSF) at <https://osf.io/r7phc/>.
